# Global burden and projections of breast cancer incidence and mortality to 2050: a comprehensive analysis of GLOBOCAN data

**DOI:** 10.3389/fpubh.2025.1622954

**Published:** 2025-10-30

**Authors:** Omar Freihat, David Sipos, Arpad Kovacs

**Affiliations:** ^1^Department of Public Health, College of Health Science, Abu Dhabi University, Abu Dhabi, United Arab Emirates; ^2^College of Health Sciences, University of Pecs, Pécs, Hungary; ^3^Department of Oncoradiology, Faculty of Medicine, University of Debrecen, Debrecen, Hungary

**Keywords:** breast cancer, global burden, incidence, mortality, projections

## Abstract

**Background:**

Breast cancer is a leading global health challenge, exhibiting significant regional disparities in incidence, mortality, and survival outcomes. This study analyzed the burden of breast cancer in 2022 and projects its future impact by 2050 using GLOBOCAN data.

**Methods:**

Incidence and mortality data for breast cancer from 2022 were analyzed across continents, age group, HDI and countries categories. The Average Annual Percent Change (AAPC) from 2018 to 2022 was calculated to project cases and deaths for 2050. Mortality-to-Incidence Ratios (MIR) were computed to assess survival disparities.

**Results:**

In 2022, Asia accounted for the highest breast cancer incidence (985,817 cases), followed by Europe (557,532) and Northern America (306,307). Africa recorded the highest mortality-to-incidence ratio (MIR) of 0.510, highlighting challenges in early detection and treatment. By 2050, global breast cancer cases are projected to exceed 6 million, with Asia, experiencing the most significant rise (2.0 million cases) followed by Africa (1.118 million cases), followed by. Mortality is expected to rise proportionally, with Asia (484,468) and Africa (390,695 deaths) and bearing the largest burden. The MIR for 2050 shows marked disparities, with Africa (0.35) and Asia (0.25) remaining elevated compared to Europe (0.20) and Northern America (0.13).

**Conclusion:**

The projected rise in breast cancer incidence and mortality highlights the urgent need for region-specific interventions. Targeted strategies focusing on early detection, improved access to treatment, and reduction of modifiable risk factors are essential, particularly in transitioning economies where disparities remain stark.

## Introduction

In 2022, breast cancer was diagnosed in 2.3 million women worldwide, resulting in 670,000 deaths. It affects women in every country and can occur at any age after puberty, with incidence rates rising with age (1). Breast cancer is the leading cause of cancer-related mortality among females. While it was once predominantly seen as a disease of developed countries, more than half of breast cancer diagnoses and two-thirds of breast cancer deaths in 2020 occurred in less developed regions of the world (2, 3).

Breast cancer incidence is strongly associated with human development. The Human Development Index (HDI), which combines measures of life expectancy, education, and wealth, serves as a more comprehensive and effective comparison between countries than income alone (4). The global age-standardized incidence rate of breast cancer in females is estimated at 48 per 100,000, ranging from below 30 per 100,000 in sub-Saharan Africa to over 70 per 100,000 in Western Europe and North America. While the relative incidence is highest in more developed regions, the larger populations in less developed regions result in over half of all breast cancer cases being diagnosed in low- and middle-income countries, posing a substantial disease burden (3).

Generally, the global burden of cancer incidence and mortality is increasing rapidly, driven by population aging and growth, as well as shifts in the prevalence and distribution of key cancer risk factors, many of which are linked to socioeconomic development (5, 6). However, recent evidence also highlights a concerning rise in early-onset breast cancer, particularly among women under 50, which accounted for the highest cancer-related DALYs in this age group globally as of 2019 (7). Studies such as Zhao et al. have documented an upward trend in early-onset breast cancer incidence, potentially linked to factors such as lifestyle changes, environmental exposures, and genetic predispositions, even as overall incidence remains elevated with age (7). This dual trend, rising cases in both younger and older populations, underscores the need for comprehensive projections that account for age-specific dynamics.

This study explores the global burden of invasive breast cancer in 2022 using GLOBOCAN estimates of cancer incidence and mortality provided by the International Agency for Research on Cancer (IARC). It also analyses geographic variations, highlighting the magnitude and distribution of the disease in 2022, and projects the future burden of breast cancer for the year 2050.

## Methods

### Study design and data sources

This study evaluates global and regional trends in breast cancer incidence and mortality using data from the GLOBOCAN 2022 database for 185 countries or territories. The database provides comprehensive statistics, including the number of new cases and deaths for primary invasive cancers of the female breast cancer. Corresponding population data for 2022 were extracted from the United Nations World Population Prospects 2022 (8). The hierarchy of methods used in compiling the cancer estimates has been described in detail elsewhere and includes short-term predictions and modeled mortality-to-incidence (M: I) ratios (9).

We provide tables and figures showcasing estimated new cases and deaths, along with two summary measures derived using direct standardization. These include age-standardized incidence and mortality rates (ASR) per 100,000 females, based on the adapted 1966 Segi World standard population for all age groups combined, and truncated ASRs for ages 15–39, 40–65, and +65. These measures enable comparisons across populations while accounting for differences in age structures. Historical incidence and mortality from 2000 to 2021 were extracted from institute of health metrics and evaluation (10). A total of 2050 predictions were made by applying 2018–2022 rates to population estimates from the United Nations Development Program (UNDP) (11). Results are presented by continent and categorized according to the six WHO continents and 185 countries and the UN’s 2020 Human Development Index (HDI), which divides countries into low, medium, high, and very high HDI levels to assess cancer burden across various stages of development. The terms “transitioning,” “emerging,” and “lower HDI countries” refer to nations with low or medium HDI, while “transitioned” or “higher HDI countries” indicate those with high or very high HDI. The Global Cancer Observatory (GCO)^1^ offers tools for tabulating and visualizing the GLOBOCAN database, facilitating analyses of the current and projected burden of female breast cancer.

### Study population

The study population includes women aged 15 years and older across all countries. Data were stratified into the following age groups: adolescents and young adults (15–39 years), adults (40–64 years), and older adults (65 years and above). The stratification was chosen to reflect established epidemiological categorizations of cancer risk and burden across the life course. Notably, the 15–39 age group is recognized by the U. S. National Cancer Institute (NCI) as defining the AYA population for cancer surveillance and research, including breast cancer (12, 13). This definition is widely adopted in global epidemiological studies, including GLOBOCAN and other international cancer statistics (13), allowing meaningful comparisons and targeted assessment of disease burden in younger populations. Moreover, these groupings allow for the analysis of age-specific trends and disparities in breast cancer incidence and mortality, moreover, ASR was presented as all ages combined, these measures allow comparisons between populations adjusted for differences in age structures.

### Analysis

The mortality-to-incidence ratio (MIR) was calculated for each HDI category to assess the relationship between incidence and mortality. MIR was determined using the formula: MIR = Mortality/Incidence. The MIR provides a measure of survival disparities, where higher values indicate poorer outcomes, reflecting challenges in early detection and access to treatment. The MIR was computed for each HDI level based on data retrieved from the Global Burden of Disease (GBD) platform.^2^^,^^3^ Historical data on incidence and mortality from previous years were utilized to establish trends. Population projections for 2050 were sourced from the United Nations’ World Population Prospects 2022 (8).^4^ These projections were used to estimate future incidence and mortality by applying growth rates to baseline data from 2022 (14).

Historical incidence and mortality from 2000 to 2021 were extracted from the Institute of Health Metrics and Evaluation (IHME). Average Annual Percent Change (AAPC) was calculated for 2018–2021 using the formula (15):


$$\text{AAPC}={\left(\frac{\mathrm{AS}R_{2021}}{\mathrm{AS}R_{2018}}\right)}^{1\backslash 3}-1$$

where the exponent 1/3 reflects the four-year period (2018–2021) and the rate is per 100,000 population. Age-standardized incidence and mortality rates (ASRs, per 100,000 population) for breast cancer were extracted from the Global Burden of Disease (GBD) database of the Institute for Health Metrics and Evaluation (IHME) for the years 2018–2021. These rates were used to calculate the average annual percent change (AAPC) to capture recent temporal trends.

The age-standardized rate (ASR) for 2050 was projected using the formula (16, 17):


$$\mathrm{AS}R_{2050}^{x}=\mathrm{AS}R_{2022}\times {\left(1+\frac{{\text{AAPC}}_{x}}{100}\right)}^{28}$$

where ASR^x^_2022_ is 2022 ASR, AAPC_x_ is the Average Annual Percent Change (central, lower, or upper bound, in %), and 28 is the number of years from 2022 to 2050. This compound growth model projects future rates with uncertainty intervals derived from IHME data.

The number of cases in 2050 was estimated using the formula (18–20):


$${\text{Cases}}_{2050}^{x}=\frac{{\mathrm{ASR}}_{2050}^{x}}{100,000}\times {\text{Population}}_{2050}$$

where Cases^x^_2050_ is the estimated case count, ASR^x^_2050_ is the projected age-standardized rate per 100,000 (central, lower, or upper bound), and Population2050 is the 2050 females’ population. The index (x) reflects uncertainty intervals derived from the Average Annual Percent Change (AAPC). This method scales the ASR to the population size.

To assess breast cancer incidence and mortality at a more granular level, we identified the top five countries within each continent based on the latest available data. This selection was determined by ranking countries according to their age-standardized incidence rates (ASIR) and age-standardized mortality rates (ASMR) per 100,000 females. The primary data source for these metrics was the GLOBOCAN 2022 database, which provides comprehensive cancer statistics compiled by the International Agency for Research on Cancer (IARC).

## Results

### Global breast cancer incidence and mortality in 2022

**Figure 1:**
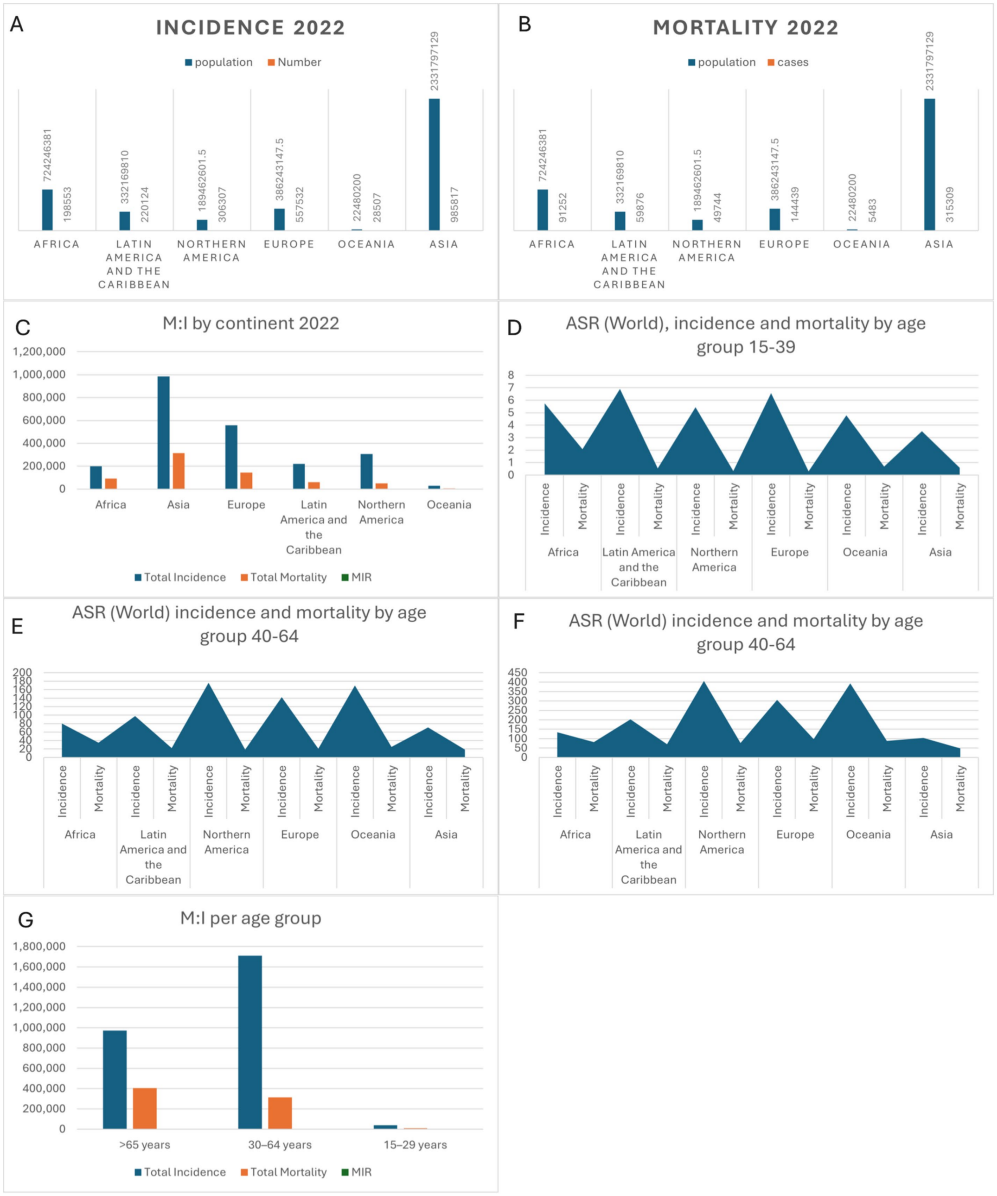
**(A–C)** Global incidence, mortality and MIR by continent in 2022. **(D–G)** incidence, mortality and MIR by age groups (15–39, 40–64, and +65).

In 2022, 2,296,840 new breast cancer cases and 666,103 deaths were recorded worldwide, with significant regional and age-based variations. Asia accounted for the highest incidence (985,817 cases, ASIR 34.34), followed by Europe (557,532 cases, ASIR 75.61) and Northern America (306,307 cases, ASIR 95.12). Latin America and the Caribbean reported 220,124 cases (ASIR 51.98), while Africa recorded 198,553 cases (ASIR 40.5). Oceania, with a smaller population, had 28,507 cases but a high ASIR of 91.48 (Table 1 and Figure 1A).

**Table 1:** Incidence and mortality 2022.

By continent				By age group		
				15–39	40–64	+65
Continent	Measure	ASR (world)	MIR	ASR (world)		
Africa	Incidence	40.5	0.46	5.76	80.28	133.55
	Mortality	19.16		2.07	34.84	82.18
Latin America and the Caribbean	Incidence	51.98	0.272	6.9	97.5	202.56
	Mortality	13.21		0.54	21.92	70.94
Northern America	Incidence	95.12	0.162	5.45	176.5	406.44
	Mortality	12.32		0.32	18.49	77.15
Europe	Incidence	75.61	0.259	6.57	142.06	305.67
	Mortality	14.55		0.31	20.67	97.42
Oceania	Incidence	91.48	0.192	4.8	169.58	393.36
	Mortality	15.43		0.69	24.75	87.13
Asia	Incidence	34.34	0.32	3.52	70.92	103.03
	Mortality	10.46		0.58	18.81	47.84
				MIR		
				0.278	0.184	0.417

Asia also recorded the most deaths (315,309, ASMR 10.46), followed by Europe (144,439, ASMR 14.55). Africa exhibited the highest mortality rate (91,252 deaths, ASMR 19.16), while Northern America (49,744 deaths, ASMR 12.32) and Oceania (5,483 deaths, ASMR 15.43) reported lower figures (Table 1 and Figure 1B).

The Mortality-to-Incidence Ratio (MIR) highlighted regional disparities. Africa’s MIR (0.460) indicated severe challenges in care, while Northern America (0.162) and Oceania (0.192) had the lowest MIRs, reflecting advanced healthcare systems (Table 1 and Figure 1C).

Age also influenced outcomes. Adolescents and young adults (15–39 years) saw the highest cases in Asia (18,180, ASIR 3.52), but Africa had the highest mortality rate (ASMR 2.07). Middle-aged adults (40–64 years) showed the highest incidence in Asia (716,882 cases), while Northern America had the highest ASIR (176.5). Older adults (65 + years) in Northern America had the highest ASIR (406.44), while Africa recorded the highest ASMR (82.18) (Table 1 and Figures 1D–F).

MIR varied by age, highest in older adults (0.417), reflecting mortality burdens. Middle-aged adults (MIR 0.184) benefitted from better survival outcomes, while adolescents faced moderate challenges (MIR 0.278). These findings underscore global disparities in breast cancer outcomes (Table 1 and Figure 1G).

### Incidence and mortality by HDI levels

**Figure 2:**
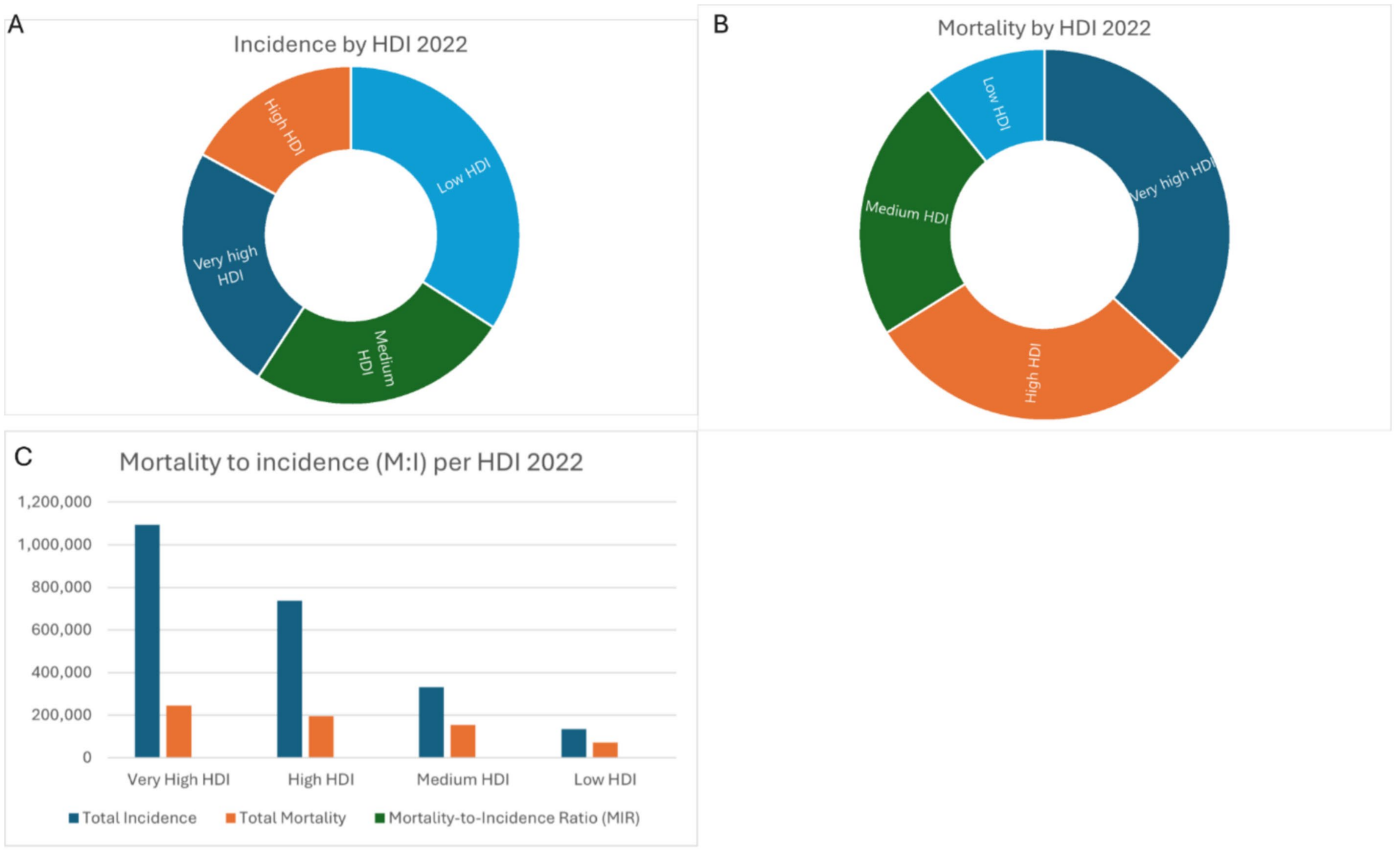
Incidence **(A)**, mortality **(B)**, and MIR **(C)** by HDI.

In 2022, breast cancer incidence varied by HDI levels. Very high HDI countries had the highest ASIR (75.6 per 100,000, 1,092,663 cases), while low HDI countries reported 34.1 per 100,000 (134,122 cases). Mortality followed a similar trend, with very high HDI countries recording the lowest ASMR (13.2 per 100,000, 245,043 deaths) and low HDI countries the highest (19.0 per 100,000, 71,458 deaths). MIR was lowest in very high HDI countries (0.224) and highest in low HDI countries (0.533), reflecting survival disparities (Table 2 and Figures 2A–C).

**Table 2:** Incidence and mortality by HDI Levels, 2022.

HDI	Cases	ASR (world)	MIR
Incidence			
Very high HDI	1,092,663	75.6	–
High HDI	737,959	38.8	–
Medium HDI	331,089	29.7	–
Low HDI	134,122	34.1	–
Mortality			
Very high HDI	245,043	13.2	–
High HDI	195,347	9.5	–
Medium HDI	153,835	14.0	–
Low HDI	71,458	19.0	–
Mortality to incidence ratio (MIR)			
Very high HDI	–	–	0.224
High HDI	–	–	0.265
Medium HDI	–	–	0.465
Low HDI	–	–	0.533

### Projections of incidence and mortality from 2018 to 2022 and 2050

Using AAPC from 2018 to 2021, breast cancer incidence and mortality were projected for 2050. Africa is expected to record 851,488 cases and 283,665 deaths, with an AAPC of 2.9% for incidence and 1.7% for mortality. Asia is projected to reach 2,729,446 cases and 706,324 deaths, with AAPC rates of 2.8 and 2.1%, respectively. Latin America and the Caribbean are estimated at 598,256 cases and 119,322 deaths, with AAPC values of 3.4% for incidence and 2.4% for mortality. Northern America projects 402,221 cases and 63,967 deaths, with AAPCs of 0.4% for incidence and 0.4% for mortality, reflecting advanced healthcare systems. Europe anticipates 460,458 cases and 129,680 deaths, with modest negative AAPCs for incidence (−0.6%) and mortality (−0.3%). Oceania, despite a smaller population, projects 77,486 cases and 13,704 deaths, with AAPC rates of 1.2% for incidence and 0.9% for mortality (Table 3).

**Table 3:** Projections of incidence and mortality from 2018 to 2021 from IHME data ASR (per 100,000), 2022 ASR (per 100,000) and 2050 projection.

Continent	Measure (ASR)	Year											
		2018	2019	2020	2021	AACP (%)	2022 (cases)	2022 ASR	2050 ASR*	2050 (cases)**	Lower UI#	Upper UI#	MIR 2050
Africa	Incidence	18.44	19	19.57	20.1	2.91	198,553	40.5	90.42	1,118,434	953,139	1,406,628	0.332
	Mortality	9.74	9.92	10.11	10.25	1.72	91,252	19.6	30.89	383,521	369,705	437,614	
Latin America and the Caribbean	Incidence	49.13	50.99	52.33	54.24	3.35	220,124	51.98	130.78	486,270	322,838	827,544	0.196
	Mortality	16.3	16.8	17.07	17.49	2.38	59,876	13.21	25.52	94,418	74,915	131,710	
Northern America	Incidence	154.41	153.75	153.55	156.48	0.44	306,307	95.12	107.56	227,298	210,784	234,107	0.129
	Mortality	30.98	30.83	30.82	31.32	0.36	49,744	12.32	13.74	28,831	26,521	29,852	
Europe	Incidence	122.57	122.15	118.93	120.24	−0.46	557,532	75.61	67.44	228,228	211,667	253,987	0.21
	Mortality	37.11	37	36.5	36.73	−0.34	144,439	14.55	13.29	47,744	44,081	53,540	
Oceania	Incidence	22.43	23.13	23.21	23.23	1.18	28,507	91.48	129.02	36,548	46,678	39,903	0.161
	Mortality	13.77	14.14	14.15	14.13	0.86	5,483	15.43	20.48	5,667	6,781	5,836	
Asia	Incidence	37.89	39.29	40.16	41.17	2.81	985,817	34.34	73.28	1,952,412	1,717,768	2,494,400	0.259
	Mortality	12.71	13.06	13.25	13.51	2.06	315,309	10.46	18.96	496,070	417,594	596,324	

### Mortality-to-incidence ratio trends (2000–2021 and 2050 projections)

**Figure 3:**
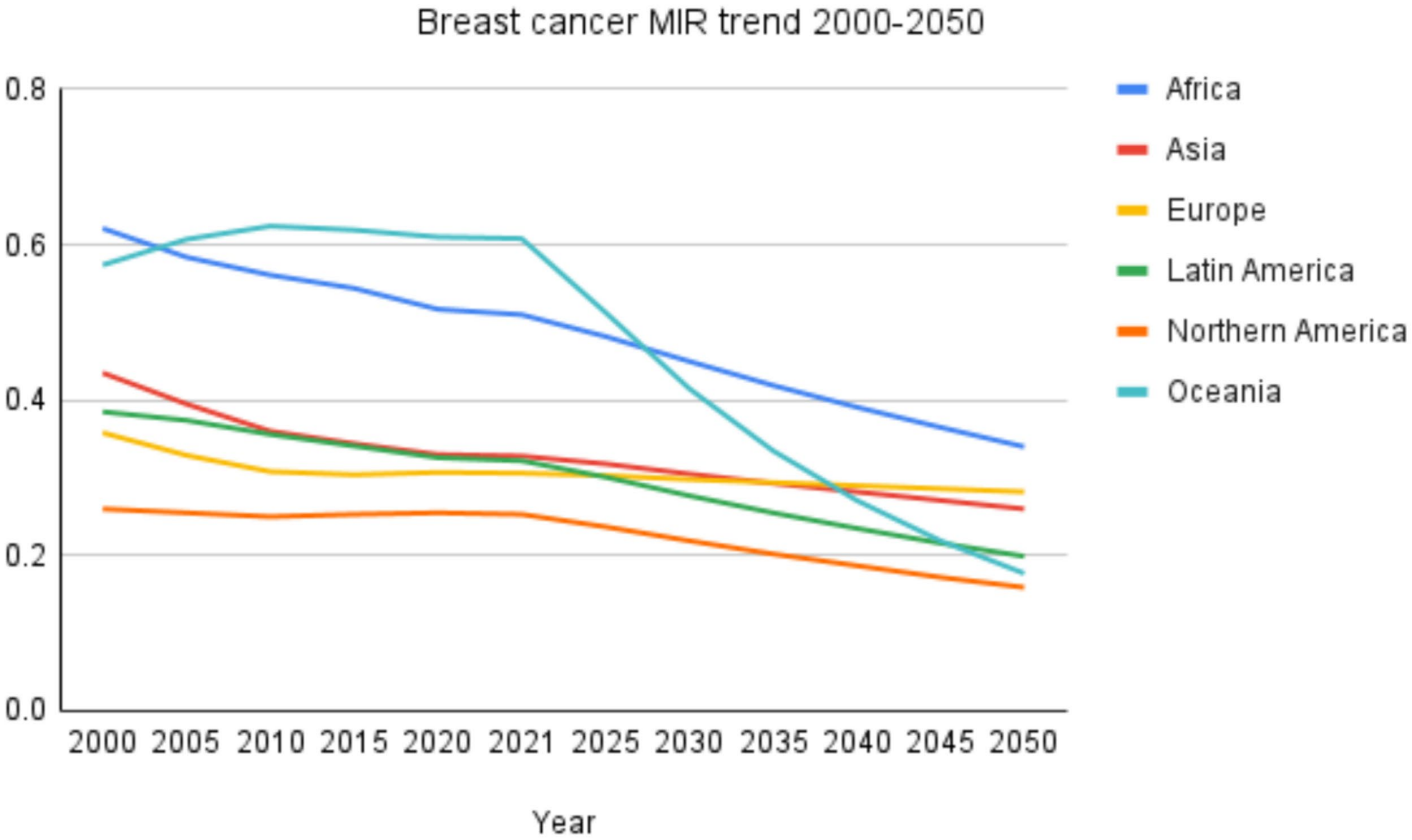
Breast cancer MIR trend 2000-2050.

From 2000 to 2021, Africa’s M: I ratio declined from 0.621 to 0.510 but remains the highest, projected to decrease further to 0.340 by 2050. Asia’s MIR fell from 0.435 in 2000 to 0.328 in 2021 and is expected to reach 0.260 by 2050. Europe showed a decline from 0.358 in 2000 to 0.306 in 2021, with a modest further reduction to 0.282 projected by 2050. Latin America and the Caribbean’s MIR decreased from 0.385 to 0.322 over 2000–2021 and is projected to drop substantially to 0.199 in 2050. Northern America consistently exhibited the lowest MIR values, with 0.260 in 2000, 0.253 in 2021, and projected at 0.159 by 2050. Oceania, which recorded the highest MIR in 2010 (0.624), stabilized at 0.608 in 2021 but is projected to decline markedly to 0.177 by 2050. These trends are summarized in Table 4 and illustrated in Figure 3.

**Table 4:** Mortality-to-incidence ratio trends (2000–2021 and 2050 projections).

Year	Africa	Asia	Europe	Latin America	Northern America	Oceania
2000	0.621	0.435	0.358	0.385	0.26	0.574
2005	0.584	0.395	0.329	0.374	0.255	0.607
2010	0.561	0.36	0.308	0.356	0.25	0.624
2015	0.544	0.344	0.304	0.341	0.253	0.619
2020	0.517	0.33	0.307	0.326	0.255	0.61
2021	0.51	0.328	0.306	0.322	0.253	0.608
2025	0.482	0.318	0.303	0.301	0.237	0.513
2030	0.45	0.305	0.298	0.277	0.219	0.415
2035	0.419	0.293	0.294	0.255	0.202	0.335
2040	0.391	0.282	0.29	0.235	0.187	0.271
2045	0.365	0.271	0.286	0.216	0.172	0.219
2050	0.34	0.26	0.21	0.196	0.129	0.162

### Country based analysis 2022

**Figure 4:**
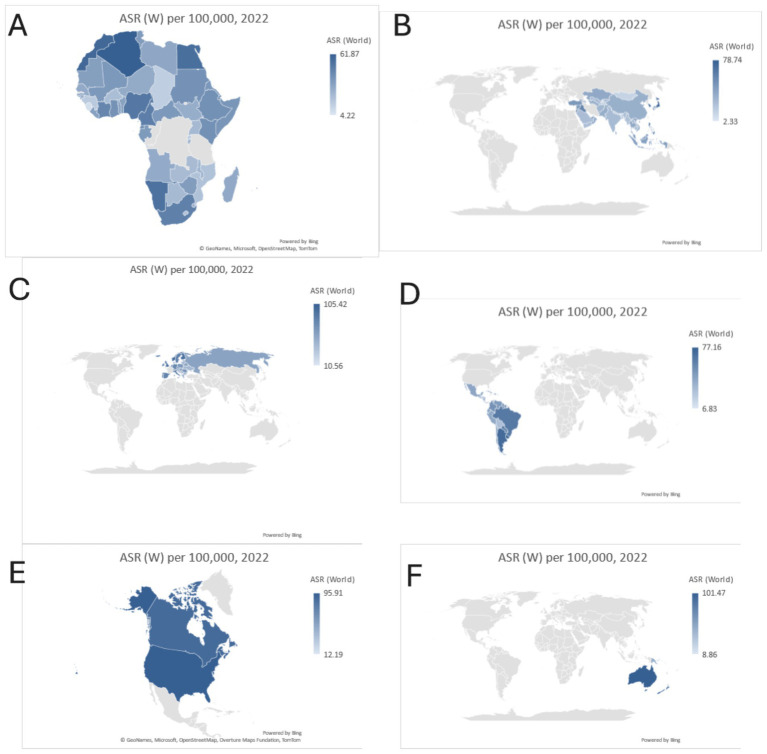
Country based analysis 2022, **(A)** Africa, **(B)** Asia, **(C)** Europe, **(D)** Latin America and the Caribbean, **(E)** Northern America, and **(F)** Oceania.

Breast cancer incidence and mortality rates vary significantly across regions. In Africa, Algeria reports the highest incidence rate (61.87 per 100,000), followed by Morocco (58.37), Namibia (53.93), Mauritius (52.68), and Nigeria (51.50). Mortality is highest in Nigeria (26.83 per 100,000), reflecting disparities in healthcare access, while Mauritius and Morocco show relatively lower mortality rates, indicating better management (Table 5 and Figure 4A).

**Table 5:** Incidence and mortality in top five countries in Africa.

Africa			Asia			Europe		
Country	Incidence ASR	Mortality ASR	Country	Incidence ASR	Mortality ASR	Country	Incidence ASR	Mortality ASR
Algeria	61.87	20.8	Israel	78.74	16.2	France	105.42	15.78
Morocco	58.37	18.07	Japan	74.39	9.68	Cyprus	104.75	18.61
Namibia	53.93	22.54	Singapore	72.61	17.82	Belgium	104.39	14.19
Mauritius	52.68	18.97	Republic of Korea	61.48	5.8	The Netherlands	101.6	14.53
Nigeria	51.5	26.83	Philippines	60.34	21.47	Luxembourg	99.7	15.09
Latin America and Caribbean			Northern America			Oceania		
France, Martinique	77.16	16.29	United States of America	95.91	12.19	Australia	101.47	12.28
Argentina	71.32	17.58	Canada	88.59	13.39	New Zealand	94.36	15.52
Uruguay	75.05	21.57	–	–	–	New Caledonia	90.18	16.81
Bahamas	64.64	31.69	–	–	–	Samoa	88.75	28.09
Brazil	63.12	13.88	–	–	–	French Polynesia	71.91	23.21

In Asia, Israel leads with an incidence rate of 78.74 per 100,000, followed by Japan (74.39) and Singapore (72.61). Mortality is highest in the Philippines (21.47 per 100,000), while Japan and the Republic of Korea exhibit significantly lower mortality (9.68 and 5.80 per 100,000), highlighting effective healthcare systems (Table 5 and Figure 4B).

Europe has some of the highest incidence rates globally, with France (105.42 per 100,000) and Cyprus (104.75) leading. Mortality rates are comparatively lower, with Cyprus reporting the highest mortality among the top countries (18.61 per 100,000). These trends emphasize the importance of continued early detection efforts. (Table 5 and Figure 4C).

In Latin America and the Caribbean, France (Martinique) shows the highest incidence (77.16 per 100,000), with the Bahamas reporting the highest mortality (31.69 per 100,000). These disparities underline the need for targeted interventions in high-risk areas (Table 5 and Figure 4D).

In Northern America, the U. S. (95.91 per 100,000) and Canada (88.59 per 100,000) exhibit high incidence rates but maintain low mortality (12.19 and 13.39 per 100,000) due to advanced healthcare infrastructure (Table 5 and Figure 4E).

Oceania reports high incidence rates, with Australia (101.47 per 100,000) and New Zealand (94.36) leading. Mortality remains relatively low in these countries (12.28 and 15.52 per 100,000), reflecting effective cancer care. Samoa exhibits the highest mortality in the region (28.09 per 100,000) (Table 5 and Figure 4F).

## Discussion

Breast cancer continues to pose a significant public health challenge globally, with substantial regional and socioeconomic disparities in incidence, mortality, and survival outcomes. The findings of this study, which assessed the global burden of breast cancer in 2022 and projected future trends to 2050, underscore the critical need for targeted interventions that address region-specific challenges. This discussion contextualizes the results within the broader scientific literature, exploring underlying causes, trends, and opportunities for improved breast cancer control worldwide.

### Geographic disparities in breast cancer burden

The geographic variation in breast cancer incidence and mortality reflects complex interactions between socioeconomic development, healthcare infrastructure, and lifestyle factors. In 2022, regions such as Northern America, Oceania, and Europe exhibited the highest age-standardized incidence rates (ASIR), consistent with findings from prior global studies (2, 9). The elevated incidence in these regions is largely attributed to widespread adoption of mammographic screening, which facilitates early detection, as well as the prevalence of known risk factors, including delayed childbearing, lower fertility rates, shorter breastfeeding durations, obesity, and alcohol consumption (21, 22).

Conversely, regions such as Africa and Asia exhibited lower ASIR but disproportionately higher mortality rates, as reflected in their elevated mortality-to-incidence ratios (MIR). These findings align with previous studies highlighting the challenges faced by low- and middle-income countries, including late-stage diagnosis, limited access to timely and effective treatment, and fragmented healthcare systems (23, 24). Recent region-specific estimates from Assessing the African burden of breast cancer (25) report around 198,300 new cases and 91,300 deaths in Africa in 2022, confirming the magnitude of burden we find. Similarly, for Asia, recent analyses confirm these upward trajectories. Burdens of Breast Cancer and Projections for 2030 in Asia (26) reported consistent rises in incidence and mortality across Southeast Asia, projecting further increases by 2030. These findings align with our 2050 projections and emphasize the growing public health challenge in rapidly transitioning economies. However, the observed decline in MIR across most regions primarily reflects the faster rise in incidence relative to mortality. While increasing incidence can be attributed to healthcare improvements such as early detection and screening, the slower decline in mortality suggests that therapeutic advances and treatment accessibility have not kept pace with diagnostic progress. This widening gap highlights ongoing disparities in cancer care, particularly in regions where survival gains remain limited despite improved detection.

### Socioeconomic and human development index impact

The burden of breast cancer is closely associated with levels of socioeconomic development, as measured by the Human Development Index (HDI). Regions with high HDI values, such as Northern America and Europe, report higher incidence rates but lower mortality due to access to organized screening programs, advanced treatment options, and improved healthcare infrastructure (9). In contrast, low- and medium-HDI regions, including parts of Africa and Asia, experience limited healthcare access, resulting in a higher proportion of late-stage diagnoses and poorer survival outcomes (27).

The increasing burden of breast cancer in transitioning economies can be attributed to the epidemiological transition characterized by changes in reproductive behaviors, urbanization, and lifestyle factors. Studies have shown that rising obesity rates, physical inactivity, and dietary changes contribute to the growing incidence of breast cancer in Low- and Middle-Income Countries (2, 28). Additionally, improvements in life expectancy and population growth further exacerbate the disease burden, as demonstrated by the projections for 2050.

### Rising incidence in transitioning countries

Consistent with our projections, Wang et al. (26) demonstrate upward ASIR trends in Asia and forecast further increases over the coming decade. The rapid rise in breast cancer incidence in transitioning regions, particularly Africa, Latin America, and parts of Asia, mirrors patterns observed in high-income countries several decades ago. This increase reflects both demographic changes, such as population growth and aging, and growing exposure to risk factors including urbanization, delayed childbearing, and reduced breastfeeding (29, 30). Evidence from sub-Saharan Africa has shown annual increases in incidence rates exceeding 5% in certain regions, underscoring the urgency for preventive and early detection measures (24).

### Mortality and survival disparities

The persistently high mortality rates observed in Africa and Asia emphasize the need for improved access to diagnosis and treatment. Studies have consistently demonstrated that late-stage presentation is a major contributor to poor survival outcomes in Low- and Middle-Income Countries. In sub-Saharan Africa, for example, up to 77% of breast cancer cases are diagnosed at advanced stages (III and IV), compared to less than 15% in high-income countries (31, 32). In a recent study by Limenih et al. estimate 5-year survival around 40%, underscoring structural challenges which our high projected mortality in Africa reflect (33). This stark contrast highlights the critical importance of implementing cost-effective early detection strategies, such as breast self-examination and clinical breast examination, in resource-limited settings (34).

In high-income regions, declining breast cancer mortality rates have been attributed to the combined impact of early detection through organized mammographic screening and advances in treatment, including targeted therapies and personalized medicine (35, 36). The experience of these regions provides valuable insights for Low- and Middle-Income Countries, where resource-stratified guidelines and evidence-based interventions can help bridge the survival gap (1, 32).

Recent regional studies reinforce that health system capacity heavily modulates outcomes. For example, Omotoso et al. (2023) report widespread limitations in sub-Saharan Africa, including inadequate diagnostic and screening infrastructure, poorly organized cancer registries, shortages of oncology specialists, high costs of care, and late presentation as key drivers of elevated mortality rates (37). Moreover, Wang and Wang emphasize that, besides late-stage diagnosis, disparities include younger age at diagnosis in Asian populations, differential subtype distributions, and limited screening and diagnostic access relative to Western settings. These factors compound system capacity issues and may partly explain why mortality does not decline as rapidly as incidence in many transitioning regions (38). Similarly, the WHO African Region assessment finds that in many low-HDI countries, over 70% of breast cancer cases are diagnosed at advanced stages (III/IV), survival rates are highly variable, and registry coverage is patchy. These system-level challenges help explain why mortality has not declined as fast as incidence, and why MIR remains high in many low- and middle-income regions (39).

### Future projections and policy implications

The projections for 2050 presented in this study highlight the growing global burden of breast cancer, with incidence and mortality expected to rise significantly in all regions. Asia is projected to remain the epicenter of the global burden due to its large population, while Africa is expected to experience the most rapid growth in both incidence and mortality. Although mortality-to-incidence ratios are projected to decline across all continents, disparities will persist, with Africa and Asia continuing to record substantially higher ratios than Northern America and Europe. These findings underscore the need for region-specific interventions that address the unique challenges faced by each continent.

For high-income regions, continued efforts to optimize screening programs, reduce modifiable risk factors (e.g., obesity and alcohol consumption), and improve treatment outcomes are essential. For low- and middle-income countries, priority should be given to early diagnosis, improved access to affordable treatments, and strengthening healthcare systems to ensure equitable care delivery (28, 40). The WHO Global Breast Cancer Initiative, which aims to reduce breast cancer mortality by 2.5% annually through early detection, diagnosis, and treatment, provides a strategic framework for achieving these goals (34).

### Strengths and limitations

A key strength of this study is the integration of recent temporal trends from IHME (2018–2021) with the most up-to-date baseline estimates from GLOBOCAN 2022, enabling robust projections of breast cancer incidence and mortality to 2050. This combined approach leverages IHME’s annualized AAPC trends to capture short-term dynamics while anchoring them to the widely recognized GLOBOCAN dataset, thereby enhancing both temporal sensitivity and global comparability. The use of continent-level stratification, along with top-performing countries, provides valuable insights into regional variations and opportunities for targeted interventions. Nevertheless, the projections remain dependent on current demographic forecasts and the assumption of stable AAPC trends, which may not fully account for future changes in risk factor prevalence, healthcare access, screening uptake, or treatment innovations, a recognized challenge in long-term forecasting. While uncertainty intervals (UIs) were calculated by propagating the lower and upper bounds of the AAPC, they remain approximations and cannot fully capture all sources of variability such as sudden policy shifts or data quality limitations. Finally, interpretation of MIR trends should be made with caution: declining MIRs may result from rising incidence rather than true reductions in mortality, reflecting diagnostic advances without equivalent treatment gains (41).

Moreover, future projections must be interpreted with caution, as emerging risk factors may significantly alter the trajectory of breast cancer incidence and mortality. Genetic predispositions (e.g., BRCA1/2 and other high-penetrance mutations) are increasingly identified through population-level genetic testing and could influence prevention and early detection strategies (42). Environmental exposures, including endocrine-disrupting chemicals, air pollution, and occupational carcinogens, are gaining recognition as contributors to breast cancer risk, particularly in rapidly industrializing regions (43, 44). In parallel, novel therapeutic approaches such as targeted therapies, immunotherapy, and precision medicine are expected to improve survival outcomes and may reduce future mortality (45, 46). Incorporating these evolving factors into future modeling frameworks will be essential to refine long-term forecasts and guide policy planning.

## Conclusion

Breast cancer remains a major global health challenge, with significant disparities in incidence, mortality, and survival outcomes across regions. The findings of this study highlight the urgent need for targeted interventions to address the growing burden, particularly in transitioning economies. By implementing evidence-based early detection strategies, improving access to treatment, and addressing modifiable risk factors, substantial progress can be made in reducing breast cancer mortality and improving outcomes worldwide.

## Data Availability

The datasets presented in this study can be found in online repositories. The names of the repository/repositories and accession number(s) can be found in the article/Supplementary material.

## Footnotes

^1^ https://gco.iarc.fr

^2^ https://www.healthdata.org/

^3^ https://vizhub.healthdata.org/

^4^ https://population.un.org/dataportal/home?df=546fe0f5-1349-4c30-af69-5c5b0a084e14
