# Supplementary material for: Global burden and projections of breast cancer incidence and mortality to 2050: a comprehensive analysis of GLOBOCAN data
Source: Front Public Health. 2025 Oct 30;13:1622954. doi: 10.3389/fpubh.2025.1622954 (PMC12611818; doi:10.3389/fpubh.2025.1622954)
Supplement: Supplementary file 1 [file Table_1.docx]

| Supplementary S1: Incidence by continent 2022 | | | | | |
| --- | --- | --- | --- | --- | --- |
| continent | population | Number | ASR (World) | Crude rate | Cumulative risk |
| Africa | 724246381 | 198553 | 40.5 | 28.2 | 4.3 |
| Latin America and the Caribbean | 332169810 | 220124 | 52.0 | 65.1 | 5.6 |
| Northern America | 189462601.5 | 306307 | 95.1 | 162.5 | 10.3 |
| Europe | 386243147.5 | 557532 | 75.6 | 144.3 | 8.1 |
| Oceania | 22480200 | 28507 | 91.5 | 130.5 | 9.9 |
| Asia | 2331797129 | 985817 | 34.3 | 43.4 | 3.7 |
| Total |  | 2296840 | 46.8 | 58.7 | 5.1 |

| Supplementary S2: Mortality by continent 2022 | | | | | |
| --- | --- | --- | --- | --- | --- |
| continent | population | Number | ASR (World) | Crude rate | Cumulative risk |
| Africa | 724246381 | 91252 | 19.2 | 13.0 | 2.1 |
| Latin America and the Caribbean | 332169810 | 59876 | 13.2 | 17.7 | 1.4 |
| Northern America | 189462601.5 | 49744 | 12.3 | 26.4 | 1.3 |
| Europe | 386243147.5 | 144439 | 14.6 | 37.4 | 1.6 |
| Oceania | 22480200 | 5483 | 15.4 | 25.1 | 1.6 |
| Asia | 2331797129 | 315309 | 10.5 | 13.9 | 1.2 |
| Total |  | 666103 | 12.7 | 17.0 | 1.4 |

| Supplementary S3: Incidence and mortality by age group (15-39) 2022 | | | | | |
| --- | --- | --- | --- | --- | --- |
| Label | | ASR (World) | Crude rate | Cumulative risk | Total |
| Africa | Incidence | 5.76 | 5.36 | 0.09 | 10132 |
|  | Mortality | 2.07 | 1.92 | 0.03 | 3631 |
| Latin America and the Caribbean | Incidence | 6.9 | 7.25 | 0.11 | 5753 |
|  | Mortality | 0.54 | 0.57 | 0.01 | 450 |
| Northern America | Incidence | 5.45 | 6.01 | 0.09 | 2190 |
|  | Mortality | 0.32 | 0.36 | 0.01 | 130 |
| Europe | Incidence | 6.57 | 7.22 | 0.1 | 4203 |
|  | Mortality | 0.31 | 0.35 | 0 | 201 |
| Oceania | Incidence | 4.8 | 5 | 0.07 | 228 |
|  | Mortality | 0.69 | 0.72 | 0.01 | 33 |
| Asia | Incidence | 3.52 | 3.67 | 0.05 | 18180 |
|  | Mortality | 0.58 | 0.6 | 0.01 | 2977 |

| Supplementary S4: Incidence and mortality by age group (30-64) | | | | | |
| --- | --- | --- | --- | --- | --- |
| Label | | ASR (World) | Crude rate | Cumulative risk | Total |
| Africa | Incidence | 80.28 | 71.92 | 2.94 | 150969 |
|  | Mortality | 34.84 | 30.78 | 1.3 | 64611 |
| Latin America and the Caribbean | Incidence | 97.5 | 96.13 | 3.6 | 141748 |
|  | Mortality | 21.92 | 21.56 | 0.84 | 31797 |
| Northern America | Incidence | 176.5 | 189.74 | 6.51 | 158421 |
|  | Mortality | 18.49 | 20.34 | 0.72 | 16982 |
| Europe | Incidence | 142.06 | 154.27 | 5.21 | 282330 |
|  | Mortality | 20.67 | 23.3 | 0.8 | 42634 |
| Oceania | Incidence | 169.58 | 172.77 | 6.25 | 15964 |
|  | Mortality | 24.75 | 25.27 | 0.94 | 2335 |
| Asia | Incidence | 70.92 | 70.29 | 2.6 | 716882 |
|  | Mortality | 18.81 | 18.64 | 0.72 | 190118 |

| Supplementary S5: Incidence and mortality by age group +65 | | | | | |
| --- | --- | --- | --- | --- | --- |
| Label | | ASR (World) | Crude rate | Cumulative risk | Total |
| Africa | Incidence | 133.55 | 133.68 |  | 37405 |
|  | Mortality | 82.18 | 82.21 |  | 23004 |
| Latin America and the Caribbean | Incidence | 202.56 | 204.89 |  | 72612 |
|  | Mortality | 70.94 | 77.95 |  | 27627 |
| Northern America | Incidence | 406.44 | 404.29 |  | 145692 |
|  | Mortality | 77.15 | 90.55 |  | 32632 |
| Europe | Incidence | 305.67 | 311.61 |  | 270991 |
|  | Mortality | 97.42 | 116.83 |  | 101604 |
| Oceania | Incidence | 393.36 | 400.81 |  | 12315 |
|  | Mortality | 87.13 | 101.38 |  | 3115 |
| Asia | Incidence | 103.03 | 102.02 |  | 250592 |
|  | Mortality | 47.84 | 49.74 |  | 122180 |

| Supplementary S6: Incidence and mortality by HDI | | | | | | | |
| --- | --- | --- | --- | --- | --- | --- | --- |
| Incidence | | | | | | | |
| Label | Number | 95% UI low | 95% UI high | Number | ASR (World) | Crude rate | Cumulative risk |
| Very HDI country | 1092663 | 1084577 | 1100809 | 1092663 | 75.6 | 131.6 | 8.2 |
| High HDI country | 737959 | 733993 | 741946 | 737959 | 38.8 | 53.9 | 4.1 |
| Medium HDI country | 331089 | 328834 | 333360 | 331089 | 29.7 | 29.8 | 3.2 |
| Low HDI country | 134122 | 131486 | 136810 | 134122 | 34.1 | 22.3 | 3.6 |
| Total | 2295833 |  |  | 2295833 | 46.8 | 58.7 | 5.1 |
| Mortality | | | | | | | |
| Label | Number | 95% UI low | 95% UI high | Number | ASR (World) | Crude rate | Cumulative risk |
| Very HDI country | 245043 | 241022 | 249131 | 245043 | 13.2 | 29.5 | 1.4 |
| High HDI country | 195347 | 193005 | 197718 | 195347 | 9.5 | 14.3 | 1.0 |
| Medium HDI country | 153835 | 152189 | 155499 | 153835 | 14.0 | 13.9 | 1.6 |
| Low HDI country | 71458 | 69305 | 73678 | 71458 | 19.0 | 11.9 | 2.1 |
| Total | 665683 |  |  | 665683 | 12.7 | 17.0 | 1.4 |

| Supplementary S7: Age standardized mortality 2022 | | | | |
| --- | --- | --- | --- | --- |
| Label | ASR (World) | Crude rate | Cumulative risk | Total |
| Africa | 19.16 | 12.97 |  | 91252 |
| Latin America and the Caribbean | 13.21 | 17.7 |  | 59876 |
| Northern America | 12.32 | 26.38 |  | 49744 |
| Europe | 14.55 | 37.39 |  | 144439 |
| Oceania | 15.43 | 25.09 |  | 5483 |
| Asia | 10.46 | 13.87 |  | 315309 |

| Supplementary S8: Age standardized incidence 2022 | | | | |
| --- | --- | --- | --- | --- |
| Label | ASR (World) | Crude rate | Cumulative risk | Total |
| Africa | 40.5 | 28.22 |  | 198553 |
| Latin America and the Caribbean | 51.98 | 65.08 |  | 220124 |
| Northern America | 95.12 | 162.46 |  | 306307 |
| Europe | 75.61 | 144.33 |  | 557532 |
| Oceania | 91.48 | 130.45 |  | 28507 |
| Asia | 34.34 | 43.36 |  | 985817 |

| Supplementary S9: Incidence and mortality 2018-2021 | | | | | | |
| --- | --- | --- | --- | --- | --- | --- |
| measure | location | age | year | val | upper | lower |
| Deaths | Africa | All ages | 2018 | 63331.77731 | 70936.5306 | 55690.0907 |
| Deaths | North America | All ages | 2018 | 57378.21782 | 60636.43796 | 51751.7029 |
| Deaths | Latin America & Caribbean - WB | All ages | 2018 | 58270.77546 | 60713.66237 | 54923.1145 |
| Deaths | Europe | All ages | 2018 | 162251.5504 | 170750.4481 | 146106.542 |
| Deaths | Asia | All ages | 2018 | 281121.7941 | 305637.9662 | 257518.466 |
| Deaths | Oceania | All ages | 2018 | 863.6651662 | 1063.129254 | 693.996113 |
| Incidence | Europe | All ages | 2018 | 535841.8458 | 557185.1575 | 498352.173 |
| Incidence | Latin America & Caribbean - WB | All ages | 2018 | 167547.3156 | 172985.2522 | 160854.641 |
| Incidence | Oceania | All ages | 2018 | 1406.692754 | 1746.157525 | 1119.80522 |
| Incidence | Africa | All ages | 2018 | 119958.9614 | 133745.4077 | 106038.519 |
| Incidence | North America | All ages | 2018 | 286015.279 | 298512.7096 | 265488.964 |
| Incidence | Asia | All ages | 2018 | 837773.5614 | 917724.99 | 761197.438 |
| Deaths | Africa | All ages | 2019 | 66097.19599 | 75254.36117 | 57834.3459 |
| Deaths | Oceania | All ages | 2019 | 908.4537712 | 1118.750844 | 744.106282 |
| Deaths | Latin America & Caribbean - WB | All ages | 2019 | 60365.38481 | 63312.01973 | 56732.6143 |
| Deaths | North America | All ages | 2019 | 57469.04612 | 60794.82353 | 51718.0333 |
| Deaths | Asia | All ages | 2019 | 291633.7806 | 319240.0325 | 265144.631 |
| Deaths | Europe | All ages | 2019 | 161854.6857 | 170596.8215 | 145393.158 |
| Incidence | Europe | All ages | 2019 | 534391.988 | 557279.9558 | 497119.984 |
| Incidence | Latin America & Caribbean - WB | All ages | 2019 | 174958.1376 | 181706.2559 | 167079.822 |
| Incidence | Oceania | All ages | 2019 | 1485.935196 | 1836.414214 | 1209.04455 |
| Incidence | Africa | All ages | 2019 | 126634.6096 | 143369.296 | 112169.451 |
| Incidence | North America | All ages | 2019 | 286561.1447 | 298969.1557 | 265937.693 |
| Incidence | Asia | All ages | 2019 | 877065.5718 | 964167.9774 | 787735.402 |
| Deaths | Africa | All ages | 2020 | 68958.90364 | 78750.23911 | 60146.729 |
| Deaths | North America | All ages | 2020 | 57739.83528 | 61270.27273 | 51757.3334 |
| Deaths | Latin America & Caribbean - WB | All ages | 2020 | 61343.95478 | 65130.76788 | 57198.3764 |
| Deaths | Europe | All ages | 2020 | 159635.4027 | 168789.9698 | 143539.342 |
| Deaths | Oceania | All ages | 2020 | 931.4144132 | 1154.507428 | 764.440602 |
| Deaths | Asia | All ages | 2020 | 298461.7178 | 329196.8583 | 270848.442 |
| Incidence | Europe | All ages | 2020 | 520096.0531 | 544676.7923 | 483902.458 |
| Incidence | Latin America & Caribbean - WB | All ages | 2020 | 179904.6103 | 190740.7486 | 169037.284 |
| Incidence | Oceania | All ages | 2020 | 1527.060314 | 1906.920063 | 1248.49109 |
| Incidence | Africa | All ages | 2020 | 133517.7982 | 152340.6265 | 116750.927 |
| Incidence | North America | All ages | 2020 | 287722.9171 | 300711.1552 | 265688.051 |
| Incidence | Asia | All ages | 2020 | 904503.1434 | 1016989.841 | 811588.665 |
| Deaths | Latin America & Caribbean - WB | All ages | 2021 | 62738.09713 | 67268.52857 | 57744.054 |
| Deaths | North America | All ages | 2021 | 58945.56551 | 62525.41275 | 52691.8209 |
| Deaths | Africa | All ages | 2021 | 71514.30287 | 81242.01501 | 62638.5916 |
| Deaths | Oceania | All ages | 2021 | 952.2985779 | 1175.932297 | 780.071941 |
| Deaths | Asia | All ages | 2021 | 306494.5885 | 340676.8981 | 276354.301 |
| Deaths | Europe | All ages | 2021 | 160388.2717 | 170874.5491 | 143198.725 |
| Incidence | Europe | All ages | 2021 | 524970.7496 | 552171.6058 | 484316.903 |
| Incidence | Latin America & Caribbean - WB | All ages | 2021 | 186492.0327 | 201390.3686 | 172198.104 |
| Incidence | Oceania | All ages | 2021 | 1565.109577 | 1961.170573 | 1279.00219 |
| Incidence | Africa | All ages | 2021 | 140209.7972 | 160211.1213 | 121833.803 |
| Incidence | North America | All ages | 2021 | 294486.5453 | 308327.5982 | 271152.057 |
| Incidence | Asia | All ages | 2021 | 934358.2314 | 1050748.435 | 837386.957 |

| Supplementary S10: Female population projections 2018-2022 and 2050 | | | | | | |
| --- | --- | --- | --- | --- | --- | --- |
| continent | 2018 | 2019 | 2020 | 2021 | 2022 | 2050 |
| Africa | 658645249 | 674884488.5 | 691265577 | 707708354.5 | 724246381 | 1236339540 |
| Latin America and the Caribbean | 322769790 | 325491083.5 | 327934147.5 | 330030710 | 332169810 | 371593523.5 |
| Northern America | 185539588.5 | 187021418 | 187995651 | 188535634 | 189462601.5 | 211061879 |
| Europe | 387470297.5 | 387820521 | 387738146 | 387185835.5 | 386243147.5 | 361129484.5 |
| Oceania | 21350225 | 21695149 | 21987371.5 | 22231049 | 22480200 | 28835031 |
| Asia | 2262768535 | 2282471976 | 2300851386 | 2316608633 | 2331797129 | 2616842354 |

| Supplementary S11: Incidence and mortality by country in Africa 2022 ASR | | |
| --- | --- | --- |
| country | Measure | ASR |
| Algeria | incidence | 61.87 |
| Algeria | mortality | 20.8 |
| Angola | incidence | 29.43 |
| Angola | mortality | 14.3 |
| Botswana | incidence | 23.03 |
| Botswana | mortality | 8.27 |
| Burundi | incidence | 25.32 |
| Burundi | mortality | 15.7 |
| Cameroon | incidence | 48.64 |
| Cameroon | mortality | 27.44 |
| Cape Verde | incidence | 23.4 |
| Cape Verde | mortality | 7.81 |
| Central African Republic | incidence | 37.72 |
| Central African Republic | mortality | 25.31 |
| Chad | incidence | 14.24 |
| Chad | mortality | 8.79 |
| Comoros | incidence | 21.5 |
| Comoros | mortality | 11.8 |
| Congo, Republic of | incidence | 26.85 |
| Congo, Republic of | mortality | 12.5 |
| Congo, Democratic Republic of | incidence | 26.45 |
| Congo, Democratic Republic of | mortality | 15.64 |
| Benin | incidence | 33.26 |
| Benin | mortality | 18.87 |
| Equatorial Guinea | incidence | 40.99 |
| Equatorial Guinea | mortality | 21.39 |
| Ethiopia | incidence | 40.81 |
| Ethiopia | mortality | 23.95 |
| Eritrea | incidence | 37.78 |
| Eritrea | mortality | 22.41 |
| Djibouti | incidence | 38.06 |
| Djibouti | mortality | 22.11 |
| Gabon | incidence | 36.41 |
| Gabon | mortality | 13.24 |
| The Republic of the Gambia | incidence | 12.12 |
| The Republic of the Gambia | mortality | 6.59 |
| Ghana | incidence | 40.19 |
| Ghana | mortality | 19.34 |
| Guinea | incidence | 15.95 |
| Guinea | mortality | 8.91 |
| Côte d'Ivoire | incidence | 45.44 |
| Côte d'Ivoire | mortality | 25.37 |
| Kenya | incidence | 40.77 |
| Kenya | mortality | 19.59 |
| Lesotho | incidence | 18.92 |
| Lesotho | mortality | 9.75 |
| Liberia | incidence | 33.57 |
| Liberia | mortality | 19.48 |
| Libya | incidence | 30.98 |
| Libya | mortality | 13.29 |
| Madagascar | incidence | 30.32 |
| Madagascar | mortality | 16.37 |
| Malawi | incidence | 25.19 |
| Malawi | mortality | 14.81 |
| Mali | incidence | 37.73 |
| Mali | mortality | 23.13 |
| Mauritania | incidence | 34.54 |
| Mauritania | mortality | 18.54 |
| Mauritius | incidence | 52.68 |
| Mauritius | mortality | 18.97 |
| Morocco | incidence | 58.37 |
| Morocco | mortality | 18.07 |
| Mozambique | incidence | 19.69 |
| Mozambique | mortality | 11.96 |
| Namibia | incidence | 53.93 |
| Namibia | mortality | 22.54 |
| Niger | incidence | 29.46 |
| Niger | mortality | 18.97 |
| Nigeria | incidence | 51.5 |
| Nigeria | mortality | 26.83 |
| Guinea-Bissau | incidence | 27.17 |
| Guinea-Bissau | mortality | 16.69 |
| France, La Réunion | incidence | 55.94 |
| France, La Réunion | mortality | 12.09 |
| Rwanda | incidence | 16.2 |
| Rwanda | mortality | 8.35 |
| Sao Tome and Principe | incidence | 19.91 |
| Sao Tome and Principe | mortality | 9.61 |
| Senegal | incidence | 29.89 |
| Senegal | mortality | 16.42 |
| Sierra Leone | incidence | 7.02 |
| Sierra Leone | mortality | 4.22 |
| Somalia | incidence | 38.62 |
| Somalia | mortality | 25.66 |
| South Africa | incidence | 47.8 |
| South Africa | mortality | 16.96 |
| Zimbabwe | incidence | 35.84 |
| Zimbabwe | mortality | 18.57 |
| South Sudan | incidence | 27.99 |
| South Sudan | mortality | 17.61 |
| Sudan | incidence | 39.94 |
| Sudan | mortality | 20.72 |
| Eswatini | incidence | 26.17 |
| Eswatini | mortality | 11.26 |
| Togo | incidence | 32.93 |
| Togo | mortality | 18.07 |
| Tunisia | incidence | 36.16 |
| Tunisia | mortality | 10.85 |
| Uganda | incidence | 23.34 |
| Uganda | mortality | 12.63 |
| Egypt | incidence | 55.4 |
| Egypt | mortality | 19.89 |
| Tanzania, United Republic of | incidence | 25.12 |
| Tanzania, United Republic of | mortality | 12.91 |
| Burkina Faso | incidence | 21.38 |
| Burkina Faso | mortality | 13.17 |
| Zambia | incidence | 22.32 |
| Zambia | mortality | 10.78 |

| Supplementary S12: Incidence and mortality by country in Asia 2022 | | |
| --- | --- | --- |
| Label | measure | ASR (World) |
| Afghanistan | Incidence | 29.44 |
| Afghanistan | Mortality | 17.92 |
| Azerbaijan | Incidence | 32.9 |
| Azerbaijan | Mortality | 11.8 |
| Bahrain | Incidence | 58.49 |
| Bahrain | Mortality | 19.67 |
| Bangladesh | Incidence | 15.18 |
| Bangladesh | Mortality | 7.55 |
| Armenia | Incidence | 39.61 |
| Armenia | Mortality | 16.08 |
| Bhutan | Incidence | 4.56 |
| Bhutan | Mortality | 2.33 |
| Brunei Darussalam | Incidence | 50.16 |
| Brunei Darussalam | Mortality | 14.28 |
| Myanmar | Incidence | 23.41 |
| Myanmar | Mortality | 10.09 |
| Cambodia | Incidence | 25.13 |
| Cambodia | Mortality | 11.17 |
| Sri Lanka | Incidence | 27.68 |
| Sri Lanka | Mortality | 11.24 |
| China | Incidence | 33.04 |
| China | Mortality | 6.1 |
| Georgia | Incidence | 49.04 |
| Georgia | Mortality | 20.64 |
| Gaza Strip and West Bank | Incidence | 46.33 |
| Gaza Strip and West Bank | Mortality | 19.71 |
| India | Incidence | 26.6 |
| India | Mortality | 13.7 |
| Indonesia | Incidence | 41.78 |
| Indonesia | Mortality | 14.35 |
| Iran (Islamic Republic of) | Incidence | 30.45 |
| Iran (Islamic Republic of) | Mortality | 11.02 |
| Iraq | Incidence | 56.91 |
| Iraq | Mortality | 23.51 |
| Israel | Incidence | 78.74 |
| Israel | Mortality | 16.2 |
| Japan | Incidence | 74.39 |
| Japan | Mortality | 9.68 |
| Kazakhstan | Incidence | 36.94 |
| Kazakhstan | Mortality | 12.31 |
| Jordan | Incidence | 59.99 |
| Jordan | Mortality | 19.32 |
| Korea (Democratic People Republic of) | Incidence | 32.25 |
| Korea (Democratic People Republic of) | Mortality | 9.84 |
| Republic of Korea | Incidence | 61.48 |
| Republic of Korea | Mortality | 5.8 |
| Kuwait | Incidence | 49.83 |
| Kuwait | Mortality | 16.99 |
| Kyrgyzstan | Incidence | 25.31 |
| Kyrgyzstan | Mortality | 8.19 |
| Lao People's Democratic Republic | Incidence | 31.63 |
| Lao People's Democratic Republic | Mortality | 12.55 |
| Lebanon | Incidence | 57.89 |
| Lebanon | Mortality | 20.39 |
| Malaysia | Incidence | 46.09 |
| Malaysia | Mortality | 19.3 |
| Maldives | Incidence | 39.84 |
| Maldives | Mortality | 13.2 |
| Mongolia | Incidence | 12.63 |
| Mongolia | Mortality | 3.19 |
| Oman | Incidence | 33.96 |
| Oman | Mortality | 13.1 |
| Nepal | Incidence | 14.35 |
| Nepal | Mortality | 7.6 |
| Pakistan | Incidence | 34.23 |
| Pakistan | Mortality | 18.6 |
| Philippines | Incidence | 60.34 |
| Philippines | Mortality | 21.47 |
| Timor-Leste | Incidence | 26.38 |
| Timor-Leste | Mortality | 10.24 |
| Qatar | Incidence | 39.03 |
| Qatar | Mortality | 11.39 |
| Saudi Arabia | Incidence | 25.28 |
| Saudi Arabia | Mortality | 7.64 |
| Singapore | Incidence | 72.61 |
| Singapore | Mortality | 17.82 |
| Viet Nam | Incidence | 37.96 |
| Viet Nam | Mortality | 14.67 |
| Syrian Arab Republic | Incidence | 46.21 |
| Syrian Arab Republic | Mortality | 20.94 |
| Tajikistan | Incidence | 19.47 |
| Tajikistan | Mortality | 7.38 |
| Thailand | Incidence | 37.44 |
| Thailand | Mortality | 11.81 |
| United Arab Emirates | Incidence | 57.05 |
| United Arab Emirates | Mortality | 15.92 |
| Türkiye | Incidence | 46.77 |
| Türkiye | Mortality | 12.52 |
| Turkmenistan | Incidence | 31.13 |
| Turkmenistan | Mortality | 15.57 |
| Uzbekistan | Incidence | 27.79 |
| Uzbekistan | Mortality | 12.98 |
| Yemen | Incidence | 25.35 |
| Yemen | Mortality | 14 |

| Supplementary S13: Incidence and mortality by country in Europe 2022 | | |
| --- | --- | --- |
| Label | Measure | ASR (World) |
| Albania | Incidence | 51.14 |
| Albania | Mortality | 14.49 |
| Austria | Incidence | 69.54 |
| Austria | Mortality | 14.26 |
| Belgium | Incidence | 104.39 |
| Belgium | Mortality | 14.19 |
| Bosnia Herzegovina | Incidence | 51.88 |
| Bosnia Herzegovina | Mortality | 14.98 |
| Bulgaria | Incidence | 52.82 |
| Bulgaria | Mortality | 15.45 |
| Belarus | Incidence | 56.57 |
| Belarus | Mortality | 13.3 |
| Croatia | Incidence | 74.64 |
| Croatia | Mortality | 11.65 |
| Cyprus | Incidence | 104.75 |
| Cyprus | Mortality | 18.61 |
| Czechia | Incidence | 72.51 |
| Czechia | Mortality | 11.69 |
| Denmark | Incidence | 95.42 |
| Denmark | Mortality | 14.08 |
| Estonia | Incidence | 63.06 |
| Estonia | Mortality | 13.23 |
| Finland | Incidence | 92.33 |
| Finland | Mortality | 11.89 |
| France (metropolitan) | Incidence | 105.42 |
| France (metropolitan) | Mortality | 15.78 |
| Germany | Incidence | 77.02 |
| Germany | Mortality | 15.81 |
| Greece | Incidence | 82.36 |
| Greece | Mortality | 14.74 |
| Hungary | Incidence | 76.38 |
| Hungary | Mortality | 16.86 |
| Iceland | Incidence | 71.06 |
| Iceland | Mortality | 17.13 |
| Ireland | Incidence | 91.45 |
| Ireland | Mortality | 17.18 |
| Italy | Incidence | 87.01 |
| Italy | Mortality | 14.83 |
| Latvia | Incidence | 65.95 |
| Latvia | Mortality | 16.29 |
| Lithuania | Incidence | 62.74 |
| Lithuania | Mortality | 14.47 |
| Luxembourg | Incidence | 99.7 |
| Luxembourg | Mortality | 15.09 |
| Malta | Incidence | 86.3 |
| Malta | Mortality | 13.8 |
| Moldova | Incidence | 46.57 |
| Moldova | Mortality | 17.45 |
| Montenegro | Incidence | 76.54 |
| Montenegro | Mortality | 23.26 |
| The Netherlands | Incidence | 101.6 |
| The Netherlands | Mortality | 14.53 |
| Norway | Incidence | 95.56 |
| Norway | Mortality | 11 |
| Poland | Incidence | 65.96 |
| Poland | Mortality | 17.39 |
| Portugal | Incidence | 88.75 |
| Portugal | Mortality | 14.53 |
| Romania | Incidence | 69.22 |
| Romania | Mortality | 16.47 |
| Russian Federation | Incidence | 57.67 |
| Russian Federation | Mortality | 13.58 |
| Serbia | Incidence | 60.5 |
| Serbia | Mortality | 18.52 |
| Slovakia | Incidence | 67.3 |
| Slovakia | Mortality | 18.1 |
| Slovenia | Incidence | 82.27 |
| Slovenia | Mortality | 15.03 |
| Spain | Incidence | 80.96 |
| Spain | Mortality | 10.56 |
| Sweden | Incidence | 81.41 |
| Sweden | Mortality | 11.86 |
| Switzerland | Incidence | 77.42 |
| Switzerland | Mortality | 12.36 |
| Ukraine | Incidence | 43.08 |
| Ukraine | Mortality | 13.17 |
| North Macedonia | Incidence | 65.28 |
| North Macedonia | Mortality | 19.49 |
| United Kingdom | Incidence | 94.03 |
| United Kingdom |  | 13.98 |

| Supplementary S14: Incidence and mortality by country in Latin America and the Caribbean 2022 | | |
| --- | --- | --- |
| Label | Measure | ASR (World) |
| Argentina | Incidence | 71.32 |
| Argentina | Mortality | 17.58 |
| Bahamas | Incidence | 64.64 |
| Bahamas | Mortality | 31.69 |
| Barbados | Incidence | 57.74 |
| Barbados | Mortality | 29.8 |
| Bolivia | Incidence | 26.78 |
| Bolivia | Mortality | 7.53 |
| Brazil | Incidence | 63.12 |
| Brazil | Mortality | 13.88 |
| Belize | Incidence | 46.75 |
| Belize | Mortality | 11.71 |
| Chile | Incidence | 38.23 |
| Chile | Mortality | 10.28 |
| Colombia | Incidence | 50.7 |
| Colombia | Mortality | 13.29 |
| Costa Rica | Incidence | 37.53 |
| Costa Rica | Mortality | 10.63 |
| Cuba | Incidence | 47.24 |
| Cuba | Mortality | 13.87 |
| Dominican Republic | Incidence | 53.39 |
| Dominican Republic | Mortality | 23.03 |
| Ecuador | Incidence | 39.46 |
| Ecuador | Mortality | 11.16 |
| El Salvador | Incidence | 39.65 |
| El Salvador | Mortality | 7.79 |
| French Guyana | Incidence | 55.71 |
| French Guyana | Mortality | 15.92 |
| France, Guadeloupe | Incidence | 66.41 |
| France, Guadeloupe | Mortality | 16.68 |
| Guatemala | Incidence | 28.35 |
| Guatemala | Mortality | 6.83 |
| Guyana | Incidence | 52.6 |
| Guyana | Mortality | 15.18 |
| Haiti | Incidence | 25.16 |
| Haiti | Mortality | 14.31 |
| Honduras | Incidence | 27.28 |
| Honduras | Mortality | 11.21 |
| Jamaica | Incidence | 71.12 |
| Jamaica | Mortality | 35.21 |
| France, Martinique | Incidence | 77.16 |
| France, Martinique | Mortality | 16.29 |
| Mexico | Incidence | 39.94 |
| Mexico | Mortality | 10.31 |
| Nicaragua | Incidence | 35.32 |
| Nicaragua | Mortality | 10.62 |
| Panama | Incidence | 41.39 |
| Panama | Mortality | 10.46 |
| Paraguay | Incidence | 58.37 |
| Paraguay | Mortality | 16.88 |
| Peru | Incidence | 39.3 |
| Peru | Mortality | 9.41 |
| Puerto Rico | Incidence | 62.32 |
| Puerto Rico | Mortality | 14.34 |
| Saint Lucia | Incidence | 51.68 |
| Saint Lucia | Mortality | 16.39 |
| Suriname | Incidence | 46.38 |
| Suriname | Mortality | 14.43 |
| Trinidad and Tobago | Incidence | 54 |
| Trinidad and Tobago | Mortality | 22.11 |
| Uruguay | Incidence | 75.05 |
| Uruguay | Mortality | 21.57 |
| Venezuela | Incidence | 47.1 |
| Venezuela | Mortality | 16.16 |

| Supplementary S15: Incidence and mortality by country in Northern America 2022 | | |
| --- | --- | --- |
| Label | Measure | ASR (World) |
| Canada | Incidence | 88.59 |
| Canada | Mortality | 13.39 |
| United States of America | Incidence | 95.91 |
| United States of America | Mortality | 12.19 |

| Supplementary S16: Incidence and mortality by country in Oceania 2022 | | |
| --- | --- | --- |
| Label | Measure | ASR (World) |
| Australia | Incidence | 101.47 |
| Australia | Mortality | 12.28 |
| Solomon Islands | Incidence | 49.18 |
| Solomon Islands | Mortality | 20.44 |
| Fiji | Incidence | 60.26 |
| Fiji | Mortality | 38.85 |
| French Polynesia | Incidence | 71.91 |
| French Polynesia | Mortality | 23.21 |
| Guam | Incidence | 44.15 |
| Guam | Mortality | 16.98 |
| New Caledonia | Incidence | 90.18 |
| New Caledonia | Mortality | 16.81 |
| Vanuatu | Incidence | 29.6 |
| Vanuatu | Mortality | 8.86 |
| New Zealand | Incidence | 94.36 |
| New Zealand | Mortality | 15.52 |
| Papua New Guinea | Incidence | 46.03 |
| Papua New Guinea | Mortality | 26.66 |
| Samoa | Incidence | 88.75 |
| Samoa | Mortality | 28.09 |

| Supplementary S17: Mortality-to-Incidence Ratio Trends (2000–2021 and 2050 Projections) | | | | | | |
| --- | --- | --- | --- | --- | --- | --- |
| Year | Africa | Asia | Europe | Latin America | Northern America | Oceania |
| 2000 | 0.621 | 0.435 | 0.358 | 0.385 | 0.26 | 0.574 |
| 2005 | 0.584 | 0.395 | 0.329 | 0.374 | 0.255 | 0.607 |
| 2010 | 0.561 | 0.36 | 0.308 | 0.356 | 0.25 | 0.624 |
| 2015 | 0.544 | 0.344 | 0.304 | 0.341 | 0.253 | 0.619 |
| 2020 | 0.517 | 0.33 | 0.307 | 0.326 | 0.255 | 0.61 |
| 2021 | 0.51 | 0.328 | 0.306 | 0.322 | 0.253 | 0.608 |
| 2050 | 0.432 | 0.334 | 0.112 | 0.144 | 0.051 | 0.049 |

|  | **Supplementary S17. Human Development Index and its components** | |  |  |  |  |  |  |  |  |  |  |  |  |  |
| --- | --- | --- | --- | --- | --- | --- | --- | --- | --- | --- | --- | --- | --- | --- | --- |
|  |  |  |  |  |  |  |  |  |  |  |  |  |  |  |  |
|  |  |  |  | **SDG3** |  | **SDG4.3** |  | **SDG4.4** |  | **SDG8.5** |  |  |  |  |  |
|  |  |  |  |  |  |  |  |  |  |  |  |  |  |  |  |
|  |  | **Human Development Index (HDI)** |  | **Life expectancy at birth** |  | **Expected years of schooling** |  | **Mean years of schooling** |  | **Gross national income (GNI) per capita** |  | **GNI per capita rank minus HDI rank** |  | **HDI rank** |  |
| **HDI rank** | **Country** | Value |  | (years) |  | (years) |  | (years) |  | (2017 PPP $) |  |  |  |  |  |
|  |  | **2022** |  | **2022** |  | **2022** | ^a^ | **2022** | ^a^ | **2022** |  | **2022** | ^b^ | **2021** |  |
|  | **VERY HIGH HUMAN DEVELOPMENT** | | | | | | | | | | | | | | |
| 1 | Switzerland | 0.967 |  | 84.3 |  | 16.6 |  | 13.9 | ^c^ | 69,433 |  | 6 |  | 1 |  |
| 2 | Norway | 0.966 |  | 83.4 |  | 18.6 | ^d^ | 13.1 | ^c^ | 69,190 |  | 6 |  | 2 |  |
| 3 | Iceland | 0.959 |  | 82.8 |  | 19.1 | ^d^ | 13.8 |  | 54,688 |  | 16 |  | 4 |  |
| 4 | Hong Kong, China (SAR) | 0.956 |  | 84.3 |  | 17.8 |  | 12.3 |  | 62,486 |  | 6 |  | 3 |  |
| 5 | Denmark | 0.952 |  | 81.9 |  | 18.8 | ^d^ | 13.0 |  | 62,019 |  | 6 |  | 8 |  |
| 5 | Sweden | 0.952 |  | 83.5 |  | 19.0 | ^d^ | 12.7 | ^c^ | 56,996 |  | 10 |  | 5 |  |
| 7 | Germany | 0.950 |  | 81.0 |  | 17.3 |  | 14.3 |  | 55,340 |  | 11 |  | 7 |  |
| 7 | Ireland | 0.950 |  | 82.7 |  | 19.1 | ^d^ | 11.7 | ^c^ | 87,468 | ^e^ | -3 |  | 9 |  |
| 9 | Singapore | 0.949 |  | 84.1 |  | 16.9 |  | 11.9 |  | 88,761 | ^e^ | -6 |  | 10 |  |
| 10 | Australia | 0.946 |  | 83.6 |  | 21.1 | ^d^ | 12.7 |  | 49,257 |  | 14 |  | 5 |  |
| 10 | Netherlands | 0.946 |  | 82.5 |  | 18.6 | ^d^ | 12.6 |  | 57,278 |  | 4 |  | 11 |  |
| 12 | Belgium | 0.942 |  | 82.3 |  | 18.9 | ^d^ | 12.5 | ^c^ | 53,644 |  | 9 |  | 13 |  |
| 12 | Finland | 0.942 |  | 82.4 |  | 19.2 | ^d^ | 12.9 | ^c^ | 49,522 |  | 11 |  | 11 |  |
| 12 | Liechtenstein | 0.942 |  | 84.7 |  | 15.5 |  | 12.4 | ^f^ | 146,673 | ^e,g^ | -11 |  | 14 |  |
| 15 | United Kingdom | 0.940 |  | 82.2 |  | 17.6 |  | 13.4 |  | 46,624 |  | 13 |  | 17 |  |
| 16 | New Zealand | 0.939 |  | 83.0 |  | 19.7 | ^d^ | 12.9 |  | 43,665 |  | 16 |  | 14 |  |
| 17 | United Arab Emirates | 0.937 |  | 79.2 |  | 17.2 |  | 12.8 |  | 74,104 |  | -11 |  | 17 |  |
| 18 | Canada | 0.935 |  | 82.8 |  | 16.0 |  | 13.9 | ^c^ | 48,444 |  | 8 |  | 16 |  |
| 19 | Korea (Republic of) | 0.929 |  | 84.0 |  | 16.5 |  | 12.6 | ^c^ | 46,026 |  | 10 |  | 20 |  |
| 20 | Luxembourg | 0.927 |  | 82.6 |  | 14.2 |  | 13.0 | ^h^ | 78,554 | ^e^ | -15 |  | 19 |  |
| 20 | United States | 0.927 |  | 78.2 |  | 16.4 |  | 13.6 |  | 65,565 |  | -11 |  | 21 |  |
| 22 | Austria | 0.926 |  | 82.4 |  | 16.4 |  | 12.3 | ^c^ | 56,530 |  | -5 |  | 22 |  |
| 22 | Slovenia | 0.926 |  | 82.1 |  | 17.4 |  | 12.9 | ^c^ | 41,587 |  | 13 |  | 24 |  |
| 24 | Japan | 0.920 |  | 84.8 |  | 15.5 |  | 12.7 |  | 43,644 |  | 9 |  | 22 |  |
| 25 | Israel | 0.915 |  | 82.6 |  | 15.0 |  | 13.4 | ^c^ | 43,588 |  | 9 |  | 26 |  |
| 25 | Malta | 0.915 |  | 83.7 |  | 15.9 |  | 12.2 |  | 44,464 |  | 5 |  | 25 |  |
| 27 | Spain | 0.911 |  | 83.9 |  | 17.8 |  | 10.6 |  | 40,043 |  | 10 |  | 28 |  |
| 28 | France | 0.910 |  | 83.2 |  | 16.0 |  | 11.7 | ^c^ | 47,379 |  | -1 |  | 27 |  |
| 29 | Cyprus | 0.907 |  | 81.9 |  | 16.2 |  | 12.4 |  | 40,137 |  | 7 |  | 29 |  |
| 30 | Italy | 0.906 |  | 84.1 |  | 16.7 |  | 10.7 |  | 44,284 |  | 1 |  | 30 |  |
| 31 | Estonia | 0.899 |  | 79.2 |  | 15.9 |  | 13.5 |  | 37,152 |  | 9 |  | 32 |  |
| 32 | Czechia | 0.895 |  | 78.1 |  | 16.3 |  | 12.9 | ^c^ | 39,945 |  | 6 |  | 31 |  |
| 33 | Greece | 0.893 |  | 80.6 |  | 20.0 | ^d^ | 11.4 |  | 31,382 |  | 20 |  | 33 |  |
| 34 | Bahrain | 0.888 |  | 79.2 |  | 16.3 |  | 11.0 |  | 48,731 |  | -9 |  | 34 |  |
| 35 | Andorra | 0.884 |  | 83.6 |  | 12.8 |  | 11.6 |  | 54,233 | ^i^ | -15 |  | 43 |  |
| 36 | Poland | 0.881 |  | 77.0 |  | 15.9 |  | 13.2 |  | 35,151 |  | 7 |  | 35 |  |
| 37 | Latvia | 0.879 |  | 75.9 |  | 16.6 |  | 13.3 | ^c^ | 32,083 |  | 13 |  | 39 |  |
| 37 | Lithuania | 0.879 |  | 74.3 |  | 16.4 |  | 13.5 |  | 38,131 |  | 2 |  | 36 |  |
| 39 | Croatia | 0.878 |  | 79.2 |  | 15.6 |  | 12.3 | ^c^ | 34,324 |  | 5 |  | 37 |  |
| 40 | Qatar | 0.875 |  | 81.6 |  | 13.3 |  | 10.1 | ^c^ | 95,944 | ^e^ | -38 |  | 41 |  |
| 40 | Saudi Arabia | 0.875 |  | 77.9 |  | 15.2 | ^j^ | 11.3 |  | 50,620 |  | -18 |  | 37 |  |
| 42 | Portugal | 0.874 |  | 82.2 |  | 16.8 |  | 9.6 |  | 35,315 |  | 0 |  | 39 |  |
| 43 | San Marino | 0.867 |  | 83.4 |  | 12.4 |  | 10.5 | ^k^ | 57,687 | ^l^ | -30 |  | 44 |  |
| 44 | Chile | 0.860 |  | 79.5 |  | 16.8 |  | 11.1 | ^c^ | 24,431 |  | 15 |  | 42 |  |
| 45 | Slovakia | 0.855 |  | 75.3 |  | 14.7 |  | 13.0 | ^c^ | 32,171 |  | 4 |  | 45 |  |
| 45 | Türkiye | 0.855 |  | 78.5 |  | 19.7 | ^d^ | 8.8 | ^c^ | 32,834 |  | 2 |  | 48 |  |
| 47 | Hungary | 0.851 |  | 75.0 |  | 15.1 |  | 12.2 |  | 34,196 |  | -2 |  | 46 |  |
| 48 | Argentina | 0.849 |  | 76.1 |  | 19.0 | ^d^ | 11.1 |  | 22,048 |  | 17 |  | 47 |  |
| 49 | Kuwait | 0.847 |  | 80.3 |  | 15.7 | ^c^ | 7.4 | ^c^ | 56,729 |  | -33 |  | 50 |  |
| 50 | Montenegro | 0.844 |  | 76.8 |  | 15.1 |  | 12.6 | ^c^ | 22,513 |  | 12 |  | 49 |  |
| 51 | Saint Kitts and Nevis | 0.838 |  | 72.0 |  | 18.4 | ^d,m^ | 10.8 | ^n^ | 28,442 |  | 3 |  | 51 |  |
| 52 | Uruguay | 0.830 |  | 78.0 |  | 17.4 |  | 9.1 | ^c^ | 22,207 |  | 12 |  | 56 |  |
| 53 | Romania | 0.827 |  | 74.1 |  | 14.5 |  | 11.4 | ^c^ | 31,641 |  | -1 |  | 52 |  |
| 54 | Antigua and Barbuda | 0.826 |  | 79.2 |  | 15.5 | ^c^ | 10.5 | ^j^ | 18,784 |  | 18 |  | 54 |  |
| 55 | Brunei Darussalam | 0.823 |  | 74.6 |  | 13.7 |  | 9.2 |  | 59,246 |  | -43 |  | 53 |  |
| 56 | Russian Federation | 0.821 |  | 70.1 |  | 15.7 | ^c^ | 12.4 |  | 26,992 |  | 1 |  | 55 |  |
| 57 | Bahamas | 0.820 |  | 74.4 |  | 11.9 | ^k^ | 12.7 | ^c^ | 32,535 |  | -9 |  | 67 |  |
| 57 | Panama | 0.820 |  | 76.8 |  | 13.2 | ^c^ | 10.7 | ^c^ | 32,029 |  | -6 |  | 57 |  |
| 59 | Oman | 0.819 |  | 73.9 |  | 13.0 |  | 11.9 |  | 32,967 |  | -13 |  | 58 |  |
| 60 | Georgia | 0.814 |  | 71.6 |  | 16.7 |  | 12.7 |  | 15,952 |  | 19 |  | 59 |  |
| 60 | Trinidad and Tobago | 0.814 |  | 74.7 |  | 14.1 | ^o^ | 11.7 | ^c^ | 22,473 |  | 3 |  | 60 |  |
| 62 | Barbados | 0.809 |  | 77.7 |  | 16.5 | ^c^ | 9.9 | ^p^ | 14,810 |  | 24 |  | 63 |  |
| 63 | Malaysia | 0.807 |  | 76.3 |  | 12.9 |  | 10.7 | ^c^ | 27,295 |  | -7 |  | 68 |  |
| 64 | Costa Rica | 0.806 |  | 77.3 |  | 16.1 | ^c^ | 8.8 |  | 20,248 |  | 2 |  | 60 |  |
| 65 | Serbia | 0.805 |  | 74.1 |  | 14.5 |  | 11.5 | ^c^ | 19,494 |  | 3 |  | 60 |  |
| 66 | Thailand | 0.803 |  | 79.7 |  | 15.6 |  | 8.8 | ^c^ | 16,887 |  | 10 |  | 69 |  |
| 67 | Kazakhstan | 0.802 |  | 69.5 |  | 14.8 |  | 12.4 | ^c^ | 22,587 |  | -6 |  | 65 |  |
| 67 | Seychelles | 0.802 |  | 71.7 |  | 13.9 |  | 11.2 |  | 28,386 |  | -12 |  | 71 |  |
| 69 | Belarus | 0.801 |  | 73.2 |  | 14.0 |  | 12.2 | ^c^ | 18,425 |  | 5 |  | 65 |  |
|  | **HIGH HUMAN DEVELOPMENT** | | | | | | | | | | | | | | |
| 70 | Bulgaria | 0.799 |  | 71.5 |  | 13.9 |  | 11.4 |  | 25,921 |  | -12 |  | 70 |  |
| 71 | Palau | 0.797 |  | 65.4 |  | 17.2 | ^k^ | 13.0 | ^k^ | 19,344 | ^l^ | -2 |  | 64 |  |
| 72 | Mauritius | 0.796 |  | 74.0 |  | 14.6 |  | 10.0 | ^p^ | 23,252 |  | -12 |  | 72 |  |
| 73 | Grenada | 0.793 |  | 75.3 |  | 16.6 | ^c^ | 9.9 | ^j^ | 13,593 |  | 18 |  | 73 |  |
| 74 | Albania | 0.789 |  | 76.8 |  | 14.5 |  | 10.1 | ^p^ | 15,293 |  | 7 |  | 74 |  |
| 75 | China | 0.788 |  | 78.6 |  | 15.2 | ^c^ | 8.1 | ^c^ | 18,025 |  | 0 |  | 74 |  |
| 76 | Armenia | 0.786 |  | 73.4 |  | 14.4 |  | 11.3 |  | 15,388 |  | 4 |  | 79 |  |
| 77 | Mexico | 0.781 |  | 74.8 |  | 14.5 |  | 9.2 |  | 19,138 |  | -7 |  | 83 |  |
| 78 | Iran (Islamic Republic of) | 0.780 |  | 74.6 |  | 14.1 |  | 10.7 | ^c^ | 14,770 |  | 10 |  | 77 |  |
| 78 | Sri Lanka | 0.780 |  | 76.6 |  | 13.6 | ^c^ | 11.2 |  | 11,899 |  | 24 |  | 76 |  |
| 80 | Bosnia and Herzegovina | 0.779 |  | 75.3 |  | 13.3 |  | 10.5 |  | 16,571 |  | -3 |  | 77 |  |
| 81 | Saint Vincent and the Grenadines | 0.772 |  | 69.0 |  | 16.3 | ^c^ | 11.0 | ^k^ | 14,049 |  | 9 |  | 80 |  |
| 82 | Dominican Republic | 0.766 |  | 74.2 |  | 13.6 |  | 9.2 | ^c^ | 18,653 |  | -9 |  | 84 |  |
| 83 | Ecuador | 0.765 |  | 77.9 |  | 14.9 |  | 9.0 |  | 10,693 |  | 25 |  | 90 |  |
| 83 | North Macedonia | 0.765 |  | 73.9 |  | 13.0 |  | 10.2 |  | 16,396 |  | -5 |  | 82 |  |
| 85 | Cuba | 0.764 |  | 78.2 |  | 14.5 |  | 10.5 | ^c^ | 7,953 | ^q^ | 40 |  | 92 |  |
| 86 | Moldova (Republic of) | 0.763 |  | 68.6 |  | 14.9 |  | 11.8 | ^c^ | 12,964 |  | 8 |  | 81 |  |
| 87 | Maldives | 0.762 |  | 80.8 |  | 12.2 | ^c^ | 7.8 | ^c^ | 18,847 |  | -16 |  | 88 |  |
| 87 | Peru | 0.762 |  | 73.4 |  | 14.8 | ^c^ | 10.0 | ^c^ | 11,916 |  | 14 |  | 86 |  |
| 89 | Azerbaijan | 0.760 |  | 73.5 |  | 12.7 |  | 10.6 | ^c^ | 15,018 |  | -7 |  | 95 |  |
| 89 | Brazil | 0.760 |  | 73.4 |  | 15.6 |  | 8.3 | ^c^ | 14,616 |  | 0 |  | 84 |  |
| 91 | Colombia | 0.758 |  | 73.7 |  | 14.4 |  | 8.9 |  | 15,014 |  | -8 |  | 89 |  |
| 92 | Libya | 0.746 |  | 72.2 |  | 14.0 | ^j^ | 7.8 | ^r^ | 19,752 |  | -25 |  | 90 |  |
| 93 | Algeria | 0.745 |  | 77.1 |  | 15.5 |  | 7.0 | ^c^ | 10,978 |  | 13 |  | 93 |  |
| 94 | Turkmenistan | 0.744 |  | 69.4 |  | 13.2 |  | 11.1 | ^c^ | 12,860 | ^l^ | 1 |  | 93 |  |
| 95 | Guyana | 0.742 |  | 66.0 |  | 13.0 | ^o^ | 8.6 | ^p^ | 35,783 |  | -54 |  | 105 |  |
| 96 | Mongolia | 0.741 |  | 72.7 |  | 14.5 | ^c^ | 9.4 |  | 10,351 |  | 15 |  | 99 |  |
| 97 | Dominica | 0.740 |  | 73.0 |  | 13.6 | ^c^ | 9.2 | ^j^ | 12,468 |  | -1 |  | 97 |  |
| 98 | Tonga | 0.739 |  | 71.3 |  | 16.3 |  | 10.9 | ^p^ | 6,360 | ^l^ | 34 |  | 95 |  |
| 99 | Jordan | 0.736 |  | 74.2 |  | 12.6 | ^c^ | 10.4 |  | 9,295 |  | 15 |  | 98 |  |
| 100 | Ukraine | 0.734 |  | 68.6 |  | 13.3 |  | 11.1 | ^p^ | 11,416 |  | 3 |  | 86 |  |
| 101 | Tunisia | 0.732 |  | 74.3 |  | 14.6 | ^c^ | 8.0 | ^c^ | 10,297 |  | 11 |  | 101 |  |
| 102 | Marshall Islands | 0.731 |  | 65.1 |  | 16.4 |  | 12.8 | ^k^ | 6,855 |  | 28 |  | 101 |  |
| 102 | Paraguay | 0.731 |  | 70.5 |  | 13.9 | ^s^ | 8.9 |  | 13,161 |  | -9 |  | 99 |  |
| 104 | Fiji | 0.729 |  | 68.3 |  | 13.8 |  | 10.4 |  | 11,234 |  | 0 |  | 110 |  |
| 105 | Egypt | 0.728 |  | 70.2 |  | 12.9 |  | 9.8 | ^c^ | 12,361 |  | -8 |  | 103 |  |
| 106 | Uzbekistan | 0.727 |  | 71.7 |  | 12.0 |  | 11.9 |  | 8,056 |  | 16 |  | 105 |  |
| 107 | Viet Nam | 0.726 |  | 74.6 |  | 13.1 | ^t^ | 8.5 | ^c^ | 10,814 |  | 0 |  | 108 |  |
| 108 | Saint Lucia | 0.725 |  | 71.3 |  | 12.7 |  | 8.6 | ^c^ | 14,778 |  | -21 |  | 109 |  |
| 109 | Lebanon | 0.723 |  | 74.4 |  | 12.1 | ^u^ | 8.6 | ^k^ | 12,313 | ^v^ | -11 |  | 104 |  |
| 110 | South Africa | 0.717 |  | 61.5 |  | 14.3 |  | 11.6 |  | 13,186 |  | -18 |  | 105 |  |
| 111 | Palestine, State of | 0.716 |  | 73.4 |  | 13.2 |  | 9.9 |  | 6,936 |  | 18 |  | 110 |  |
| 112 | Indonesia | 0.713 |  | 68.3 |  | 14.0 | ^c^ | 8.6 |  | 12,046 |  | -12 |  | 113 |  |
| 113 | Philippines | 0.710 |  | 72.2 |  | 12.8 |  | 9.0 | ^c^ | 9,059 |  | 5 |  | 118 |  |
| 114 | Botswana | 0.708 |  | 65.9 |  | 11.4 |  | 10.4 |  | 14,842 |  | -29 |  | 124 |  |
| 115 | Jamaica | 0.706 |  | 70.6 |  | 12.5 | ^c^ | 9.2 | ^c^ | 9,695 |  | -2 |  | 114 |  |
| 116 | Samoa | 0.702 |  | 72.6 |  | 12.4 |  | 11.4 | ^c^ | 4,970 |  | 25 |  | 112 |  |
| 117 | Kyrgyzstan | 0.701 |  | 70.5 |  | 13.0 |  | 12.0 | ^c^ | 4,782 |  | 28 |  | 116 |  |
| 118 | Belize | 0.700 |  | 71.0 |  | 12.4 |  | 8.8 |  | 9,242 |  | -3 |  | 115 |  |
|  | **MEDIUM HUMAN DEVELOPMENT** | | | | | | | | | | | | | | |
| 119 | Venezuela (Bolivarian Republic of) | 0.699 |  | 71.1 |  | 13.5 | ^k^ | 9.6 | ^k^ | 6,184 | ^w^ | 14 |  | 120 |  |
| 120 | Bolivia (Plurinational State of) | 0.698 |  | 64.9 |  | 15.0 |  | 9.8 |  | 7,988 |  | 3 |  | 119 |  |
| 120 | Morocco | 0.698 |  | 75.0 |  | 14.6 |  | 6.1 |  | 7,955 |  | 4 |  | 122 |  |
| 122 | Nauru | 0.696 |  | 64.0 |  | 12.6 | ^c^ | 9.2 | ^j^ | 14,939 |  | -38 |  | 117 |  |
| 123 | Gabon | 0.693 |  | 65.7 |  | 12.4 | ^c^ | 9.6 |  | 11,194 |  | -18 |  | 123 |  |
| 124 | Suriname | 0.690 |  | 70.3 |  | 11.0 |  | 8.4 | ^c^ | 12,310 |  | -25 |  | 121 |  |
| 125 | Bhutan | 0.681 |  | 72.2 |  | 13.1 | ^c^ | 5.8 | ^c^ | 10,625 | ^v^ | -15 |  | 125 |  |
| 126 | Tajikistan | 0.679 |  | 71.3 |  | 10.9 | ^c^ | 11.3 | ^p^ | 4,807 |  | 18 |  | 125 |  |
| 127 | El Salvador | 0.674 |  | 71.5 |  | 11.9 | ^s^ | 7.2 |  | 8,886 |  | -7 |  | 127 |  |
| 128 | Iraq | 0.673 |  | 71.3 |  | 12.2 | ^t^ | 6.8 | ^p^ | 9,092 |  | -11 |  | 128 |  |
| 129 | Bangladesh | 0.670 |  | 73.7 |  | 11.9 |  | 7.4 |  | 6,511 |  | 2 |  | 130 |  |
| 130 | Nicaragua | 0.669 |  | 74.6 |  | 12.6 | ^s^ | 7.3 |  | 5,427 |  | 4 |  | 129 |  |
| 131 | Cabo Verde | 0.661 |  | 74.7 |  | 11.5 | ^c^ | 6.1 | ^k^ | 7,601 |  | -4 |  | 132 |  |
| 132 | Tuvalu | 0.653 |  | 64.9 |  | 12.1 | ^c^ | 10.6 | ^c^ | 4,754 |  | 15 |  | 131 |  |
| 133 | Equatorial Guinea | 0.650 |  | 61.2 |  | 12.1 | ^j^ | 8.3 | ^j^ | 10,663 |  | -24 |  | 133 |  |
| 134 | India | 0.644 |  | 67.7 |  | 12.6 |  | 6.6 |  | 6,951 |  | -6 |  | 135 |  |
| 135 | Micronesia (Federated States of) | 0.634 |  | 70.9 |  | 12.6 | ^j^ | 7.3 | ^j^ | 3,709 |  | 18 |  | 134 |  |
| 136 | Guatemala | 0.629 |  | 68.7 |  | 10.8 | ^c^ | 5.7 | ^c^ | 8,996 |  | -17 |  | 136 |  |
| 137 | Kiribati | 0.628 |  | 67.7 |  | 11.8 |  | 9.1 | ^k^ | 3,440 |  | 21 |  | 137 |  |
| 138 | Honduras | 0.624 |  | 70.7 |  | 10.0 | ^s^ | 7.3 | ^c^ | 5,272 |  | 2 |  | 138 |  |
| 139 | Lao People's Democratic Republic | 0.620 |  | 69.0 |  | 10.2 |  | 5.9 | ^p^ | 7,745 |  | -13 |  | 140 |  |
| 140 | Vanuatu | 0.614 |  | 70.5 |  | 11.8 | ^c^ | 7.2 | ^j^ | 3,244 |  | 21 |  | 141 |  |
| 141 | Sao Tome and Principe | 0.613 |  | 68.8 |  | 12.7 | ^o^ | 5.9 | ^c^ | 4,054 |  | 8 |  | 143 |  |
| 142 | Eswatini (Kingdom of) | 0.610 |  | 56.4 |  | 14.9 | ^c^ | 5.7 |  | 8,392 |  | -21 |  | 142 |  |
| 142 | Namibia | 0.610 |  | 58.1 |  | 11.8 | ^x^ | 7.2 | ^p^ | 9,200 |  | -26 |  | 139 |  |
| 144 | Myanmar | 0.608 |  | 67.3 |  | 12.1 | ^c^ | 6.5 | ^p^ | 4,038 |  | 6 |  | 145 |  |
| 145 | Ghana | 0.602 |  | 63.9 |  | 11.6 |  | 6.4 | ^p^ | 5,380 |  | -10 |  | 144 |  |
| 146 | Kenya | 0.601 |  | 62.1 |  | 11.4 | ^x^ | 7.7 |  | 4,808 |  | -3 |  | 147 |  |
| 146 | Nepal | 0.601 |  | 70.5 |  | 12.6 |  | 4.5 | ^c^ | 4,026 |  | 5 |  | 149 |  |
| 148 | Cambodia | 0.600 |  | 69.9 |  | 11.6 | ^k^ | 5.2 |  | 4,291 |  | 0 |  | 147 |  |
| 149 | Congo | 0.593 |  | 63.1 |  | 12.4 | ^c^ | 8.3 | ^p^ | 2,903 |  | 14 |  | 146 |  |
| 150 | Angola | 0.591 |  | 61.9 |  | 12.2 |  | 5.8 | ^x^ | 5,328 |  | -11 |  | 150 |  |
| 151 | Cameroon | 0.587 |  | 61.0 |  | 13.4 | ^c^ | 6.5 | ^p^ | 3,681 |  | 3 |  | 152 |  |
| 152 | Comoros | 0.586 |  | 63.7 |  | 13.0 | ^c^ | 6.2 | ^y^ | 3,261 |  | 8 |  | 151 |  |
| 153 | Zambia | 0.569 |  | 61.8 |  | 11.0 | ^y^ | 7.3 | ^p^ | 3,157 |  | 9 |  | 154 |  |
| 154 | Papua New Guinea | 0.568 |  | 66.0 |  | 11.1 | ^x^ | 4.9 | ^p^ | 3,710 |  | -2 |  | 155 |  |
| 155 | Timor-Leste | 0.566 |  | 69.1 |  | 13.2 | ^x^ | 6.0 | ^x^ | 1,629 |  | 24 |  | 153 |  |
| 156 | Solomon Islands | 0.562 |  | 70.7 |  | 10.3 | ^c^ | 5.9 | ^j^ | 2,273 |  | 14 |  | 155 |  |
| 157 | Syrian Arab Republic | 0.557 |  | 72.3 |  | 7.4 | ^k^ | 5.7 | ^k^ | 3,594 | ^z^ | -2 |  | 157 |  |
| 158 | Haiti | 0.552 |  | 63.7 |  | 11.1 | ^j^ | 5.6 | ^p^ | 2,802 |  | 6 |  | 158 |  |
| 159 | Uganda | 0.550 |  | 63.6 |  | 11.5 | ^x^ | 6.2 | ^c^ | 2,241 |  | 12 |  | 160 |  |
| 159 | Zimbabwe | 0.550 |  | 59.4 |  | 11.0 | ^c^ | 8.8 | ^c^ | 2,079 |  | 15 |  | 159 |  |
| **LOW HUMAN DEVELOPMENT** | | | | | | | | | | | | | | | |
| 161 | Nigeria | 0.548 |  | 53.6 |  | 10.5 |  | 7.6 |  | 4,755 |  | -15 |  | 162 |  |
| 161 | Rwanda | 0.548 |  | 67.1 |  | 11.4 |  | 4.9 |  | 2,317 |  | 8 |  | 163 |  |
| 163 | Togo | 0.547 |  | 61.6 |  | 13.0 | ^c^ | 5.6 | ^c^ | 2,214 |  | 9 |  | 160 |  |
| 164 | Mauritania | 0.540 |  | 64.7 |  | 8.1 |  | 4.8 | ^p^ | 5,344 |  | -26 |  | 164 |  |
| 164 | Pakistan | 0.540 |  | 66.4 |  | 7.9 | ^c^ | 4.4 | ^c^ | 5,374 |  | -27 |  | 165 |  |
| 166 | Côte d'Ivoire | 0.534 |  | 58.9 |  | 10.1 |  | 4.2 | ^p^ | 5,376 |  | -30 |  | 166 |  |
| 167 | Tanzania (United Republic of) | 0.532 |  | 66.8 |  | 8.6 |  | 5.6 | ^c^ | 2,578 |  | -1 |  | 167 |  |
| 168 | Lesotho | 0.521 |  | 53.0 |  | 11.1 | ^c^ | 7.5 | ^c^ | 2,709 |  | -3 |  | 168 |  |
| 169 | Senegal | 0.517 |  | 67.9 |  | 9.1 |  | 2.9 | ^c^ | 3,464 |  | -12 |  | 170 |  |
| 170 | Sudan | 0.516 |  | 65.6 |  | 8.5 | ^c^ | 3.9 |  | 3,515 |  | -14 |  | 169 |  |
| 171 | Djibouti | 0.515 |  | 62.9 |  | 8.0 | ^c^ | 3.9 | ^k^ | 4,875 |  | -29 |  | 170 |  |
| 172 | Malawi | 0.508 |  | 62.9 |  | 11.5 | ^c^ | 5.2 |  | 1,432 |  | 10 |  | 172 |  |
| 173 | Benin | 0.504 |  | 60.0 |  | 10.3 |  | 3.1 | ^p^ | 3,406 |  | -14 |  | 173 |  |
| 174 | Gambia | 0.495 |  | 62.9 |  | 9.0 | ^x^ | 4.5 |  | 2,090 |  | -1 |  | 174 |  |
| 175 | Eritrea | 0.493 |  | 66.6 |  | 7.3 | ^c^ | 5.1 | ^j^ | 1,957 | ^z^ | 2 |  | 174 |  |
| 176 | Ethiopia | 0.492 |  | 65.6 |  | 9.9 | ^c^ | 2.4 | ^c^ | 2,369 |  | -8 |  | 176 |  |
| 177 | Liberia | 0.487 |  | 61.1 |  | 10.5 |  | 5.3 | ^p^ | 1,330 |  | 8 |  | 177 |  |
| 177 | Madagascar | 0.487 |  | 65.2 |  | 9.2 | ^c^ | 4.6 |  | 1,464 |  | 4 |  | 177 |  |
| 179 | Guinea-Bissau | 0.483 |  | 59.9 |  | 10.5 | ^o^ | 3.7 |  | 1,880 |  | -1 |  | 179 |  |
| 180 | Congo (Democratic Republic of the) | 0.481 |  | 59.7 |  | 9.6 | ^c^ | 7.2 | ^p^ | 1,080 |  | 9 |  | 180 |  |
| 181 | Guinea | 0.471 |  | 59.0 |  | 10.2 | ^c^ | 2.4 | ^c^ | 2,404 |  | -14 |  | 182 |  |
| 182 | Afghanistan | 0.462 |  | 62.9 |  | 10.7 | ^c^ | 2.5 |  | 1,335 | ^z^ | 2 |  | 181 |  |
| 183 | Mozambique | 0.461 |  | 59.6 |  | 10.7 | ^c^ | 3.9 |  | 1,219 |  | 4 |  | 183 |  |
| 184 | Sierra Leone | 0.458 |  | 60.4 |  | 9.0 | ^o^ | 3.5 | ^c^ | 1,613 |  | -4 |  | 184 |  |
| 185 | Burkina Faso | 0.438 |  | 59.8 |  | 8.1 |  | 2.3 | ^c^ | 2,037 |  | -9 |  | 185 |  |
| 186 | Yemen | 0.424 |  | 63.7 |  | 7.9 | ^k^ | 2.8 | ^r^ | 1,106 | ^l^ | 2 |  | 186 |  |
| 187 | Burundi | 0.420 |  | 62.0 |  | 10.0 | ^c^ | 3.3 | ^c^ | 712 |  | 5 |  | 187 |  |
| 188 | Mali | 0.410 |  | 59.4 |  | 7.0 | ^c^ | 1.6 |  | 2,044 |  | -13 |  | 188 |  |
| 189 | Chad | 0.394 |  | 53.0 |  | 8.2 | ^c^ | 2.3 | ^c^ | 1,389 |  | -6 |  | 189 |  |
| 189 | Niger | 0.394 |  | 62.1 |  | 7.2 | ^c^ | 1.3 | ^p^ | 1,283 |  | -3 |  | 190 |  |
| 191 | Central African Republic | 0.387 |  | 54.5 |  | 7.3 | ^c^ | 4.0 | ^p^ | 869 |  | 0 |  | 191 |  |
| 192 | South Sudan | 0.381 |  | 55.6 |  | 5.6 | ^c^ | 5.7 | ^aa^ | 691 | ^l^ | 1 |  | 192 |  |
| 193 | Somalia | 0.380 |  | 56.1 |  | 7.6 | ^j^ | 1.9 |  | 1,072 |  | -3 |  | .. |  |
|  | **OTHER COUNTRIES OR TERRITORIES** | | | | | | | | | | | | | | |
|  | Korea (Democratic People's Rep. of) | .. |  | 73.6 |  | .. |  | .. |  | .. |  | .. |  | .. |  |
|  | Monaco | .. |  | 86.9 | ^ab^ | 18.7 | ^c,d^ | .. |  | .. |  | .. |  | .. |  |
|  |  |  |  |  |  |  |  |  |  |  |  |  |  |  |  |
|  | **Human development groups** | | | | | | | | | | | | | | |
|  | Very high human development | 0.902 |  | 79.3 |  | 16.6 |  | 12.3 |  | 44,958 |  | — |  | — |  |
|  | High human development | 0.764 |  | 75.2 |  | 14.5 |  | 8.6 |  | 15,484 |  | — |  | — |  |
|  | Medium human development | 0.640 |  | 68.0 |  | 12.3 |  | 6.7 |  | 6,444 |  | — |  | — |  |
|  | Low human development | 0.517 |  | 61.6 |  | 9.3 |  | 4.7 |  | 3,186 |  | — |  | — |  |
|  |  |  |  |  |  |  |  |  |  |  |  |  |  |  |  |
|  | **Developing countries** | 0.694 |  | 70.5 |  | 12.5 |  | 7.6 |  | 11,125 |  | — |  | — |  |
|  |  |  |  |  |  |  |  |  |  |  |  |  |  |  |  |
|  | **Regions** | | | | | | | | | | | | | | |
|  | Arab States | 0.704 |  | 71.3 |  | 11.9 |  | 7.8 |  | 14,391 |  | — |  | — |  |
|  | East Asia and the Pacific | 0.766 |  | 76.2 |  | 14.5 |  | 8.2 |  | 16,138 |  | — |  | — |  |
|  | Europe and Central Asia | 0.802 |  | 73.6 |  | 15.5 |  | 10.6 |  | 19,763 |  | — |  | — |  |
|  | Latin America and the Caribbean | 0.763 |  | 73.7 |  | 14.8 |  | 9.0 |  | 15,109 |  | — |  | — |  |
|  | South Asia | 0.641 |  | 68.4 |  | 11.9 |  | 6.6 |  | 6,972 |  | — |  | — |  |
|  | Sub-Saharan Africa | 0.549 |  | 60.6 |  | 10.3 |  | 6.0 |  | 3,666 |  | — |  | — |  |
|  |  |  |  |  |  |  |  |  |  |  |  |  |  |  |  |
|  | **Least developed countries** | 0.542 |  | 64.9 |  | 10.1 |  | 5.0 |  | 3,006 |  | — |  | — |  |
|  | **Small island developing states** | 0.730 |  | 71.6 |  | 12.6 |  | 8.6 |  | 16,379 |  | — |  | — |  |
|  |  |  |  |  |  |  |  |  |  |  |  |  |  |  |  |
|  | **Organisation for Economic Co-operation and Development** | 0.906 |  | 80.1 |  | 16.6 |  | 12.2 |  | 46,318 |  | — |  | — |  |
|  |  |  |  |  |  |  |  |  |  |  |  |  |  |  |  |
|  | **World** | 0.739 |  | 72.0 |  | 13.0 |  | 8.7 |  | 17,254 |  | — |  | — |  |
|  |  |  |  |  |  |  |  |  |  |  |  |  |  |  |  |
|  | **Notes** |  |  |  |  |  |  |  |  |  |  |  |  |  |  |
|  | a. Data refer to 2022 or the most recent year available. | |  |  |  |  |  |  |  |  |  |  |  |  |  |
|  | b. Based on countries for which a Human Development Index value is calculated. | | |  |  |  |  |  |  |  |  |  |  |  |  |
|  | c. Updated by HDRO based on data from UNESCO Institute for Statistics (2023). | | |  |  |  |  |  |  |  |  |  |  |  |  |
|  | d. In calculating the HDI value, expected years of schooling is capped at 18 years. | | |  |  |  |  |  |  |  |  |  |  |  |  |
|  | e. In calculating the HDI value, GNI per capita is capped at $75,000. | |  |  |  |  |  |  |  |  |  |  |  |  |  |
|  | f. Updated by HDRO using mean years of schooling trend of Austria and data from UNESCO Institute for Statistics (2023) | | | | | |  |  |  |  |  |  |  |  |  |
|  | g. Estimated using the purchasing power parity (PPP) rate and projected growth rate of Switzerland. | | | |  |  |  |  |  |  |  |  |  |  |  |
|  | h. Updated by HDRO based on data from UNESCO Institute for Statistics (2023) and OECD (2023). | | | |  |  |  |  |  |  |  |  |  |  |  |
|  | i. Estimated using the PPP rate of Spain. |  |  |  |  |  |  |  |  |  |  |  |  |  |  |
|  | j. Based on HDRO estimates using cross-country regression. | |  |  |  |  |  |  |  |  |  |  |  |  |  |
|  | k. Updated by HDRO based on data from UNESCO Institute for Statistics (2023) and estimates using cross-country regression. | | | | | |  |  |  |  |  |  |  |  |  |
|  | l. HDRO estimate based on data from World Bank (2023), United Nations Statistics Division (2023) and IMF (2023). | | | | | |  |  |  |  |  |  |  |  |  |
|  | m. Refers to 2015 based on UNESCO Institute for Statistics (2023). | |  |  |  |  |  |  |  |  |  |  |  |  |  |
|  | n. Refers to 2015 based on HDRO estimates using cross-country regression. | |  |  |  |  |  |  |  |  |  |  |  |  |  |
|  | o. Updated by HDRO based on data from UNESCO Institute for Statistics (2023) and United Nations Children's Fund (UNICEF) Multiple Indicator Cluster Surveys for various years. | | | | | | | | | |  |  |  |  |  |
|  | p. Updated by HDRO based on data from UNESCO Institute for Statistics (2023) and Barro and Lee (2018). | | | | |  |  |  |  |  |  |  |  |  |  |
|  | q. HDRO estimate based on cross-country regression and the projected growth rate from United Nations Statistics Division (2023) and UN DESA (2023). | | | | | | | |  |  |  |  |  |  |  |
|  | r. Updated by HDRO based on data from Barro and Lee (2018) and estimates using cross-country regression. | | | | |  |  |  |  |  |  |  |  |  |  |
|  | s. Updated by HDRO based on data from UNESCO Institute for Statistics (2023) and SEDLAC (CEDLAS and The World Bank) (2023). | | | | | | |  |  |  |  |  |  |  |  |
|  | t. Updated by HDRO based on data from United Nations Children's Fund (UNICEF) Multiple Indicator Cluster Surveys for various years. | | | | | | |  |  |  |  |  |  |  |  |
|  | u. Updated by HDRO based on data from United Nations Children's Fund (UNICEF) Multiple Indicator Cluster Surveys for various years and estimates using cross-country regression. | | | | | | | | | |  |  |  |  |  |
|  | v. HDRO estimate based on data from World Bank (2023) and IMF (2023). | |  |  |  |  |  |  |  |  |  |  |  |  |  |
|  | w. IMF (2023). |  |  |  |  |  |  |  |  |  |  |  |  |  |  |
|  | x. Updated by HDRO based on data from UNESCO Institute for Statistics (2023) and ICF Macro Demographic and Health Surveys for various years. | | | | | | | |  |  |  |  |  |  |  |
|  | y. Updated by HDRO based on data from ICF Macro Demographic and Health Surveys for various years. | | | |  |  |  |  |  |  |  |  |  |  |  |
|  | z. HDRO estimate based on data from World Bank (2023), United Nations Statistics Division (2023) and UN DESA (2023). | | | | | |  |  |  |  |  |  |  |  |  |
|  | aa. Refers to 2008 based on UNESCO Institute for Statistics (2023). | |  |  |  |  |  |  |  |  |  |  |  |  |  |
|  | ab. In calculating the HDI value, life expectancy is capped at 85 years. | |  |  |  |  |  |  |  |  |  |  |  |  |  |
|  |  |  |  |  |  |  |  |  |  |  |  |  |  |  |  |
|  | **Definitions** |  |  |  |  |  |  |  |  |  |  |  |  |  |  |
|  | **Human Development Index (HDI):** A composite index measuring average achievement in three basic dimensions of human development—a long and healthy life, knowledge and a decent standard of living. See *Technical note 1* at http://hdr.undp.org/sites/default/files/hdr2023_technical_notes.pdf for details on how the HDI is calculated. | | | | | | | | | | | | | | |
|  | **Life expectancy at birth:** Number of years a newborn infant could expect to live if prevailing patterns of age-specific mortality rates at the time of birth stay the same throughout the infant’s life. | | | | | | | | | | |  |  |  |  |
|  | **Expected years of schooling:** Number of years of schooling that a child of school entrance age can expect to receive if prevailing patterns of age-specific enrolment rates persist throughout the child’s life. | | | | | | | | | | | |  |  |  |
|  | **Mean years of schooling:** Average number of years of education received by people ages 25 and older, converted from education attainment levels using official durations of each level. | | | | | | | | | |  |  |  |  |  |
|  | **Gross national income (GNI) per capita:** Aggregate income of an economy generated by its production and its ownership of factors of production, less the incomes paid for the use of factors of production owned by the rest of the world, converted to international dollars using PPP rates, divided by midyear population. | | | | | | | | | | | | | | |
|  | **GNI per capita rank minus HDI rank:** Difference in ranking by GNI per capita and by HDI value. A negative value means that the country is better ranked by GNI than by HDI value. | | | | | | | | | |  |  |  |  |  |
|  | **HDI rank for 2021:** Ranking by HDI value for 2021, calculated using the same most recently revised data available in 2023 that were used to calculate HDI values for 2021. | | | | | | | | | |  |  |  |  |  |
|  |  |  |  |  |  |  |  |  |  |  |  |  |  |  |  |
|  | **Main data sources** |  |  |  |  |  |  |  |  |  |  |  |  |  |  |
|  | Columns 1 and 7: HDRO calculations based on data from Barro and Lee (2018), IMF (2023), UNDESA (2022) (2023), UNESCO Institute for Statistics (2023), United Nations Statistics Division (2023) and World Bank (2023). | | | | | | | | | | | | |  |  |
|  | Column 2: UNDESA (2022). |  |  |  |  |  |  |  |  |  |  |  |  |  |  |
|  | Column 3: CEDLAS and The World Bank (2023), ICF Macro Demographic and Health Surveys, UNESCO Institute for Statistics (2023) and UNICEF Multiple Indicator Cluster Surveys. | | | | | | | | | |  |  |  |  |  |
|  | Column 4: Barro and Lee (2018), ICF Macro Demographic and Health Surveys, OECD (2023), UNESCO Institute for Statistics (2023) and UNICEF Multiple Indicator Cluster Surveys. | | | | | | | | | |  |  |  |  |  |
|  | Column 5: IMF (2023), UNDESA (2023), United Nations Statistics Division (2023) andWorld Bank (2023). | | | |  |  |  |  |  |  |  |  |  |  |  |
|  | Column 6: Calculated based on data in columns 1 and 5. | |  |  |  |  |  |  |  |  |  |  |  |  |  |
